# Complete mitochondrial genomes of *Anopheles stephensi* and *An. dirus* and comparative evolutionary mitochondriomics of 50 mosquitoes

**DOI:** 10.1038/s41598-017-07977-0

**Published:** 2017-08-09

**Authors:** You-Jin Hao, Yi-Lin Zou, Yi-Ran Ding, Wen-Yue Xu, Zhen-Tian Yan, Xu-Dong Li, Wen-Bo Fu, Ting-Jing Li, Bin Chen

**Affiliations:** 1 Chongqing Key Laboratory of Vector Insects; Institute of Entomology and Molecular Biology, Chongqing Normal University, Chongqing, 401331 China; 2 The Department of Pathogenic Biology, Third Military Medical University, Chongqing, 400038 China

## Abstract

To better understand the phylogeny and evolution of mosquitoes, the complete mitochondrial genome (mitogenome) of *Anopheles stephensi* and *An. dirus* were sequenced and annotated, and a total of 50 mosquito mitogenomes were comparatively analyzed. The complete mitogenome of *An. stephensi* and *An. dirus* is 1,5371 bp and 1,5406 bp long, respectively. The main features of the 50 mosquito mitogenomes are conservative: 13 protein-coding genes (PCGs), two ribosomal RNA genes, 22 transfer RNA genes, positive AT-skew and negative GC-skew. The gene order *trnA*-*trnR* in ancestral insects is rearranged. All tRNA genes have the typical clover leaf secondary structure but *tRNA* ^*Ser*^. The control regions are highly variable in size. PCGs show signals of purifying selection, but evidence for positive selection in *ND2*, *ND4* and *ND6* is found. Bayesian and Maximum Likelihood phylogenetic analyses based on all PCG nucleotides produce an identical tree topology and strongly support the monophyly of subgenera *Cellia*, *Anopheles*, *Keterszia* and *Nyssorhynchus*, the sister relationship of the subgenera *Nyssorhynchus* and *Keterszia*, and *Cellia* and *Anopheles*. The most recent ancestor of the genus *Anopheles* and Culicini + Aedini exited ~145 Mya ago. This is the first comprehensive study of mosquito mitogenomes, which are effective for mosquito phylogeny at various taxonomic levels.

## Introduction

Mosquitoes (Diptera: Culicidae) are the most important group of medical and veterinary insects, and they are world-widely distributed in temperate and tropical regions^1^. The females of many mosquito species may transmit devastating pathogens and/or parasites through blood-sucking, including malaria, dengue, yellow fever, and encephalitis, which causes millions of human deaths every year^2^. Due to the medical importance, an accurate and reliable taxonomy and phylogeny construction of mosquito species are essential for our understanding of the ecology, life history strategy and disease transmission efficiency, and the design and implementation of effective control measures of mosquitoes.

Morphology-based classification is time-consuming and not always sufficient for identification of closely related species or cryptic species with overlapping geographical distributions. Therefore, multidisciplinary approaches, such as morphological characters, isoenzyme, and various molecular markers, have been adopted for mosquito taxonomy and phylogeny^3–15^. Currently, the family Culicidae is classified into two recognized subfamilies, Anophelinae and Culicinae. Subfamily Anophelinae harbors three genera (485 species) and Culicinae has 109 genera that are further divided into 11 tribes (3061 species)^1^. Though many efforts have been made on the phylogeny of genus *Anopheles* or more broadly across the family Culicidae, topological conflicts and/or discrepancies were frequently recovered from different datasets and inferring methods. Most previous studies supported that subfamily Anophelinae was monophyletic and formed the basal lineage of other Culicidae, and genus *Chagasia* presented a basal lineage of other Anophelinae species^1, 16, 17^. However, the phylogenetic relationships of genus *Bironella*, subgenera *Lophopodomyia* and *Stethomyia* within this subfamily were still problematic^5^. Within genus *Nysorhynchus*, the complete taxonomic picture for Albitarsis Complex is not yet clear, because there are conflicting tree topologies^18^. The *An. punctulatus* (AP) Group of mosquitoes, the major vectors of malaria and filariasis in the South West Pacific areas, currently contains at least 13 sibling species^19^. However, their phylogeny are not well resolved and sometimes are contradictory^20, 21^. Furthermore, a recent molecular phylogeny based on mitochondrial gene *COI* revealed that subgenera *Anopheles* and *Pyretophorus* were not monophyletic, because *An. sinensis* (*Anopheles* subgenus) and *An. epiroticus* (Pyretophorus series) were claded into subfamily Culicinae and Neomyzomyia series, respectively^22^. This result is a real challenge to the traditional taxonomy at the subgenus level. Although considerable advances in our understanding of phylogenetic relationships of mosquito lineages, the definition and identification of a specie or cryptic species complex are still problematic or remain limited.

Complete mitochondrial genome (mitogenome) has been widely used for molecular evolution, phylogenetics, phylogeography and population genetics due to its maternal inheritance, simple genome organization, and the ability to provide more phylogenetic information than individual genes^23, 24^. Using the complete mitogenome sequences in insect phylogeny has produced some remarkable results in Diptera^25^, Orthoptera^26^, Hymenoptera^27^ and Heteroptera^28^. However, it frequently produced high incongruence with the nuclear and morphological data to elucidate the relationships between orders or at higher levels in some insect groups^26–28^. Since the first mosquito mitogenome of *An. gambiae* was published^29^, the availability of mosquito mitogenome sequences is growing as a result of recent advances in sequencing strategies. However, only few studies focused on phylogeny using the complete genome sequences. Nevertheless, inconsistent phylogenies were also observed, largely due to different taxa sampling, dataset types and inferring methods. Phylogeny of 26 *Anopheles* species based on Maximum Likelihood analysis of the concatenated nine protein-coding genes recovered *An. atroparvus* + *An. quadrimaculatus* as a monophyletic clade^30^. More robust studies including more species will be really needed to better resolve this issue by using more reasonable molecular markers and rational inferring methods. Hua *et al*.^31^ recovered the monophyly of series Neomyzomia + (Pyretophorus + Myzomyia), whereas a more recent study supported the monophyly of series Pyretophorus + (Neomyzomia + Myzomyia)^30^. Therefore, how mitochondrial datasets and inferring methods affect the phylogenetic reconstruction of mosquitoes need to be further evaluated.

In the present study, we sequenced and annotated the complete mitogenomes of *An. stephensi* and *An*. *dirus*, the subgenus *Cellia*, which are of crucial importance in malaria transmission throughout the South-East Asian and Southern China. More importantly, we systematically compared 50 mosquito mitogenomes with aims to answer the following questions: i) does the positive selection act on protein-coding genes in different species or ecotypes of species at the mitogenomic level? ii) how do the mitochondrial datasets and inferring methods affect the phylogenetic reconstruction? iii) when did the major lineages in family Culicidae diverge during the evolution?

## Results and Discussions

### General features of *An. stephensi* and *An. dirus* mitogenomes

**Figure 1:**
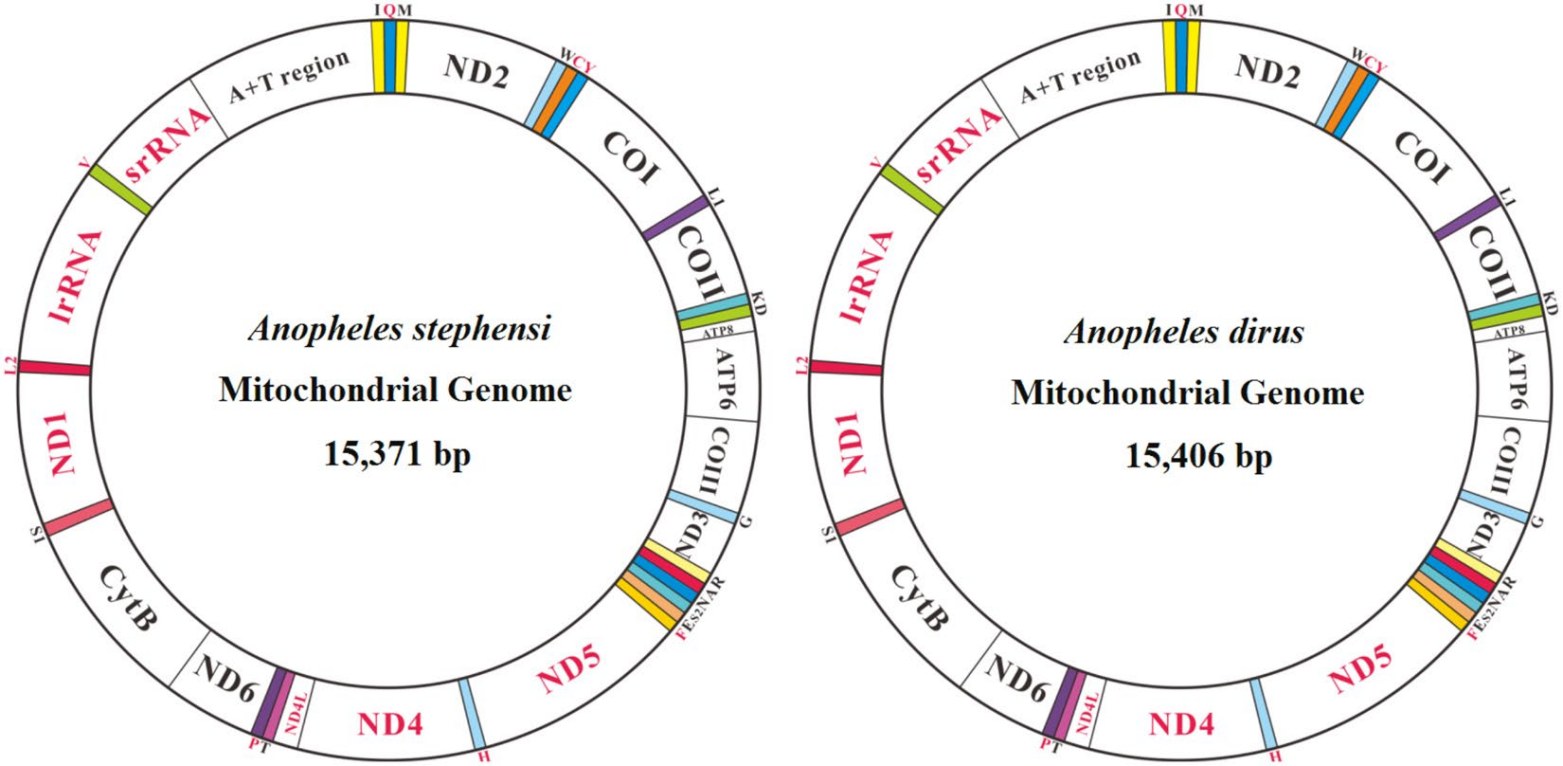
Mitochondrial genome structure of *Anopheles stephensi* and *An. dirus*. The color-filled blocks indicate tRNAs, while the un-filled white blocks denote protein-coding genes, rRNA and control regions. The protein-coding genes, rRNA and control regions with black name are located on majority strand, whereas those with red names are on minority strand.

The complete mitogenome of *An. stephensi* (KM 899887) and *An. dirus* (KM 899888) is a typical circular, double-stranded molecule with the length of 15,371 bp and 15,406 bp, respectively. Each contains a conserved set of 37 genes, including 13 protein-coding genes (PCGs), large and small ribosomal genes (*rrL* and *rrS*), 22 transfer RNA (*tRNA*) genes and a control region (also known as the AT-rich region) (Fig. 1). 23 and 14 genes are located on the majority strand (J-strand) and the minority strand (N-strand), respectively (Fig. 1). The gene order of *trnR*-*trnA* is the same as in other mosquito mitogenomes, which is a reverse order of *trnA-trnR* in ancestral insect mitogenome^32^. The gene order is a feature of mitogenome that can provide important evidence to establish evolutionary relationships among taxa at low and/or high taxonomic level^33–35^. More mosquito mitogenome sequences will be helpful to clarify whether this inversion pattern represents an evolutionary event in family Culicidae.

### Comparative analyses of 50 mosquito mitogenomes

**Figure 2:**
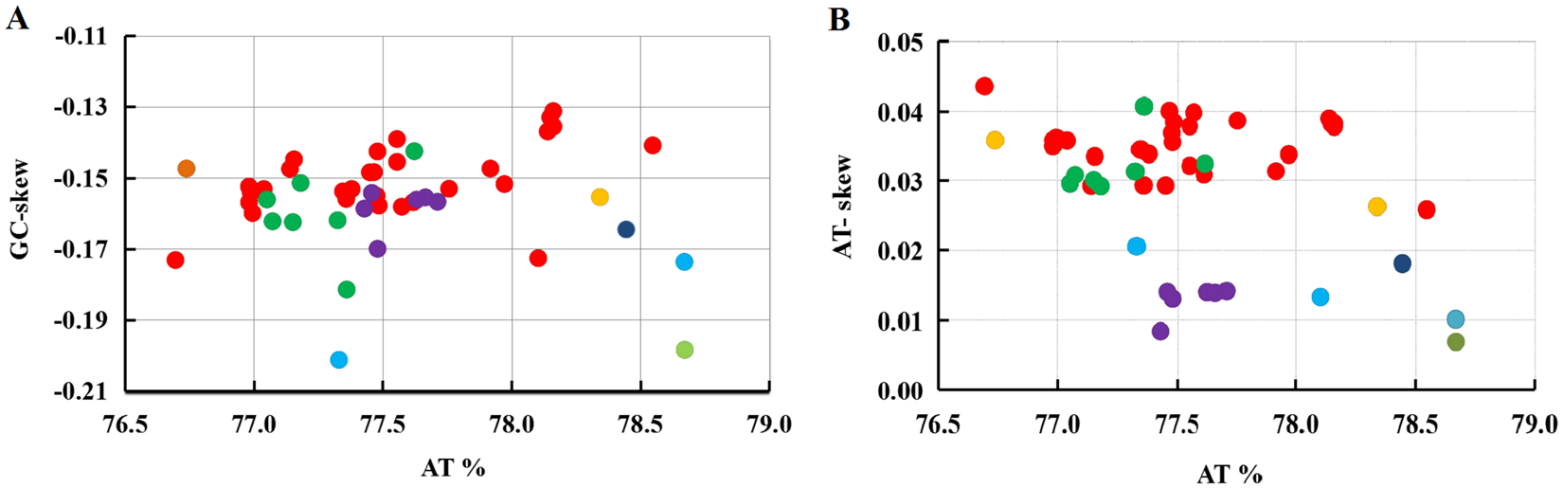
Comparative analysis of AT content, AT- and GC-skew in 50 mosquito mitogenomes. Red, orange, green, light blue, purple, blue and light green dots represent mitogenomes from the subgenus *Cellia*, *Anopheles*, *Nyssorhynchus*, *Aedes*, *Culex*, *Kertezia* and *Armigeres*, respectively.

Like other insect mitogenome sequences, base composition of mosquito mitogenomes is heterogeneous among species (Fig. 2; Supplementary Table 3). The AT content of the complete sequences excluding the control regions ranges from 76.7% for *An. christyi* to 78.7% for *Ae. notoscriptus*. All mitogenomes exhibit a positive AT-skew with the range of variation from 0.01 for subgenus *Culex* and *Ar. subalbatus* to 0.044 for *An. christyi*. However, all mitogenomes display negative GC-skews ranging from −0.2 for *Ae. aegypti* to −0.13 for *An. punctulatus* in Papua New Guinea (PNG)(Fig. 2). Insect mitogenomes exhibit a distinct strand-based nucleotide composition bias, which was thought to be due to either replication-mutation or transcription-associated mutation^24^.

AT content for all PCGs ranges from 75.3% for *An. christyi* and 79.1% for *An. minimus*. ATN, TTG, GTT and GTG are used as canonical start codons for invertebrate mitogenomes^36^. Most mosquito PCGs use ATN as the start codon, while gene *ND5* in 39 species uses GTG as the start codon, which has been considered as a common feature across various organisms^37^. All *COI* genes use a TCG start codon, which is non-canonical start codon for mitochondrial genes. Non-canonical start codon TCG in *COI* gene was previously observed in *Polystoechotes punctatus* and *Ascaloptynx appendiculatus* ^38^. Stop codons for almost all PCGs are invariable complete and incomplete stop codon T or TA, which was observed in all metazoan mitogenomes^39^ and can be completed to the full stop codon TAA through post-transcriptional modification^40^. The third codon position has a higher AT content than the first or the second position. Purifying selection against deleterious mutations is expected to be less severe on third codon position, thus higher AT content is possibly associated with the bias usage of synonymous codon.

**Figure 3:**
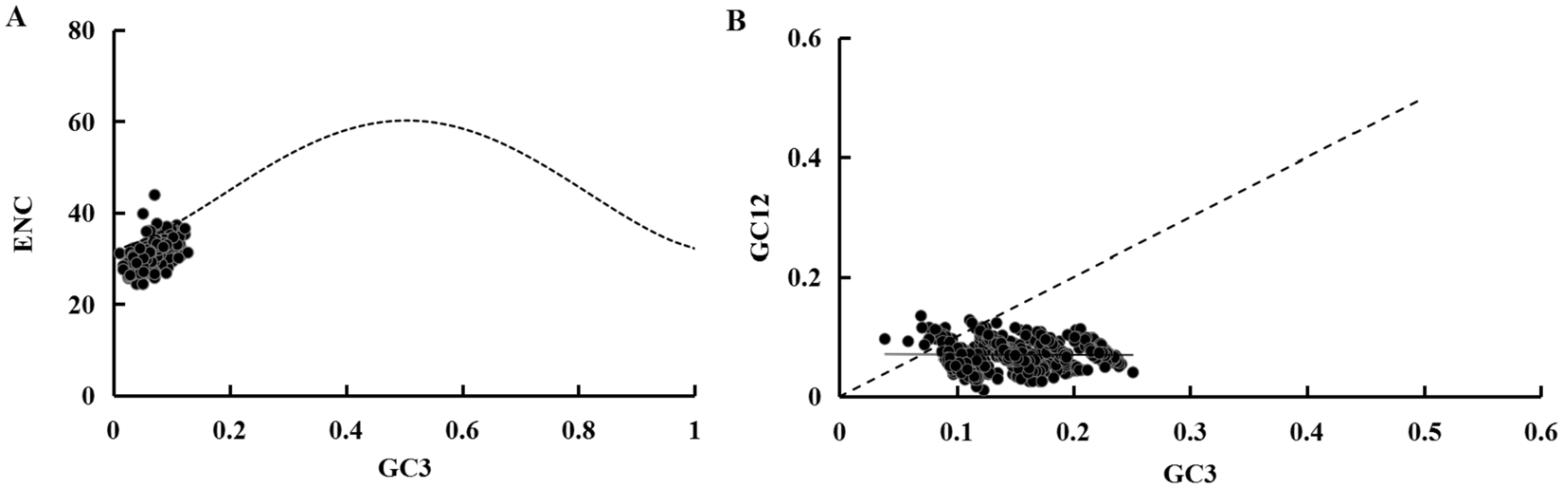
Evaluation of codon bias in 50 mosquito mitogenomes. (**A**) ENC-GC3 plot. The standard curve shows the functional relation between ENC and GC3 under mutation pressure without selection. Points on or close to the curve indicate codon use bias caused by mutation pressure; otherwise, they are affected by natural selection or other factors. (**B**) Neutrality plot of GC12 versus GC3. GC12 indicates the average value of GC content in the first and second position of the codons (GC1 and GC2); GC3 refers to the GC content in the third position.

Codon usage bias can be caused by many factors, including gene function, recombination, mutation bias, GC composition, gene length, codon position, environmental stress, population size and others^41^. Codons ending with A or U are used more frequently than those ending with CG or GC, which is a common feature in many dipteran insects. Effective number of codon (ENC) values for all PCGs range from 24.4 to 43.9, reflecting a strong codon bias. ENC-GC3 plot showed that most of the values are not close to the standard curve (Fig. 3A; Supplementary Fig. 1), indicating that not only mutation but also other factors, such as natural selection and/or translational selection, are likely to be involved in shaping the codon bias in mosquito mitogenomes. The neutrality plot revealed that all PCGs have a narrow GC3 distribution and there is no significant correlation between GC12 and GC3 (*Y* = −0.028x + 0.155, *R* ^2^ = 0.001, *P* = 0.739) (Fig. 3B). Our result addresses an important selection process that the codon bias is mainly dominated by natural selection, and mutational pressure only lightly affects the usage bias.

Hierarchical clustering of codon frequencies showed that about 50% codons are used in a bias pattern (Supplementary Fig. 2). However, the clustering result does not accurately reflect the phylogeny of mosquitoes inferred from data PCG123 below. This result will contribute to our understanding of codon usage patterns and provide better insights into their evolutionary relationships.

Most tRNAs can be folded into the typical clover-leaf structure except tRNA^*Ser*^, in which the dihydrouracil (DHU) arm is absent. The lack of a DHU arm in this gene has been commonly observed across metazoan mitogenomes^42^ (Supplementary Fig. 3). Notably, tRNA^*Leu*(*CUN*)^ uses UUR as the anticodon rather than CUN, which might be driven by the natural selection to adapt to the codon use bias. It is worthy to note that the terminal CCA is absent in all tRNAs, which is speculated to be edited by CCA-adding enzymes. Pairwise genetic distance (p-distance, pDis and maximum- likelihood distance, MLdis) and base difference (BDps) allows the level of conservation and the possible understanding of the substitution patterns in mosquito *tRNAs*. Our results showed that the *tRNA* ^*Val*^ is the most conserved one (BDps = 0.0945, MLdis = 0.007 ± 0.001 and pDis = 0.009 ± 0.003), whereas *tRNA* ^*Leu*(*CUN*)^ is the most variable one (BDps = 9.6, MLdis = 0.18 ± 0.124 and pDis = 0.13 ± 0.0083) (Supplementary Table 4). A total of 1472 base substitutions, ranging from 20 in *tRNA* ^*Val*^ to 245 in *tRNA* ^*Leu*(*CUN*)^, were detected and mapped on their secondary structures (Supplementary Fig. 3). Most variations including base substitutions and indels were found in loops, the T_Ψ_C (thymidine pseudouridine cytidine) and DHU arms. Due to the substitution heterogeneity, a perfect correspondence does not exist between the percentage of stem positions involved in the base change and the global percentage of base substitution in a single tRNA. A total of 645 BCNS (base change number in a stem) including 347 FCBC (fully compensatory base change), 51HCBC (hemi-compensatory base change), and 247 mismatches were identified in all tRNAs (Supplementary Fig. 3). Due to suffering greater mutation pressures, mitochondrial tRNAs accumulated more deleterious mutation relative to their nuclear tRNA counterparts^43^. Although mitochondrial mRNA editing was observed in some eukaryote taxa^44^, it has not been reported in mosquito mitogenomes. Therefore, we speculated that the mismatched stems could be edited by template-dependent RNA editing mechanism.

**Figure 4:**
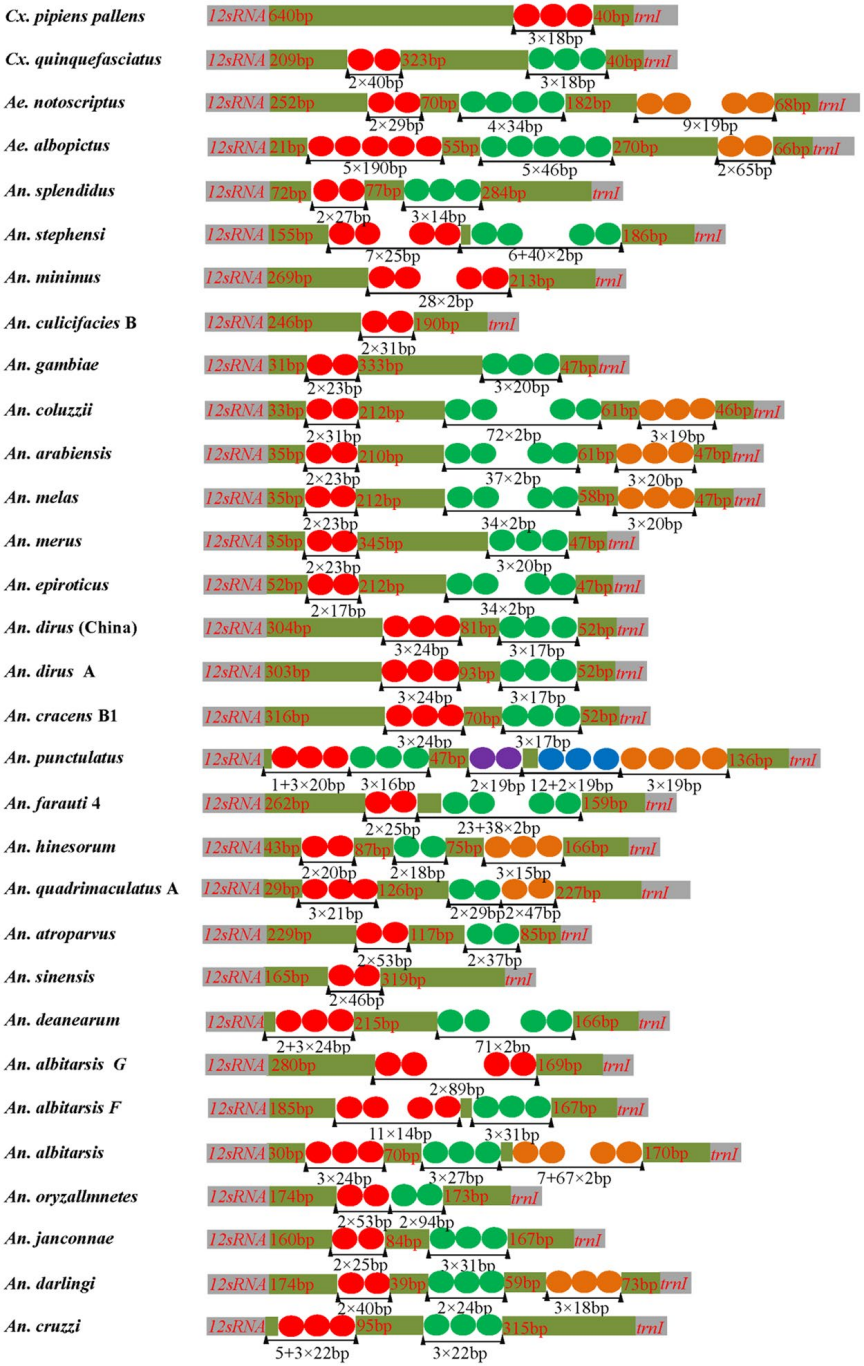
The structural organization of the control region of 30 fully completed mosquito mitogenomes. The location and copy number of tandem repeats are shown by colored circle. Non-repeat regions are indicated by colored box with sequence size inside.

The control region, known for the initiation of replication in vertebrates and invertebrates^45^, is located between *rrnS* and *tRNA* ^*Ile*^ with a varying size. The conserved motif 5′CCCCTA3′ followed by a 15–27 bp poly-T stretch was identified in the control regions of 31 mosquito mitogenomes. This motif was putatively involved in the origin of the light strand replication. *Ae. notoscriptus*, *An. dirus* China, *An. cracens*, and *An. dirus* A use the motif 5′ATTGTA3′, whereas *Ae. albopictus* uses the motif 5′TTACTA3′. In 24 mosquito mitogenomes, tandem repeat sequences harbor two repeat unit types and are interrupted by a non-coding region (Fig. 4). Overall, control regions of mosquito mitogenomes showed a distinct sequence and structural characteristic, which may be taxon-specific and can be potentially used as a genetic marker for evolutionary and/or population genetic studies of mosquito species.

### Evidence of positive selection on protein-coding genes

**Figure 5:**
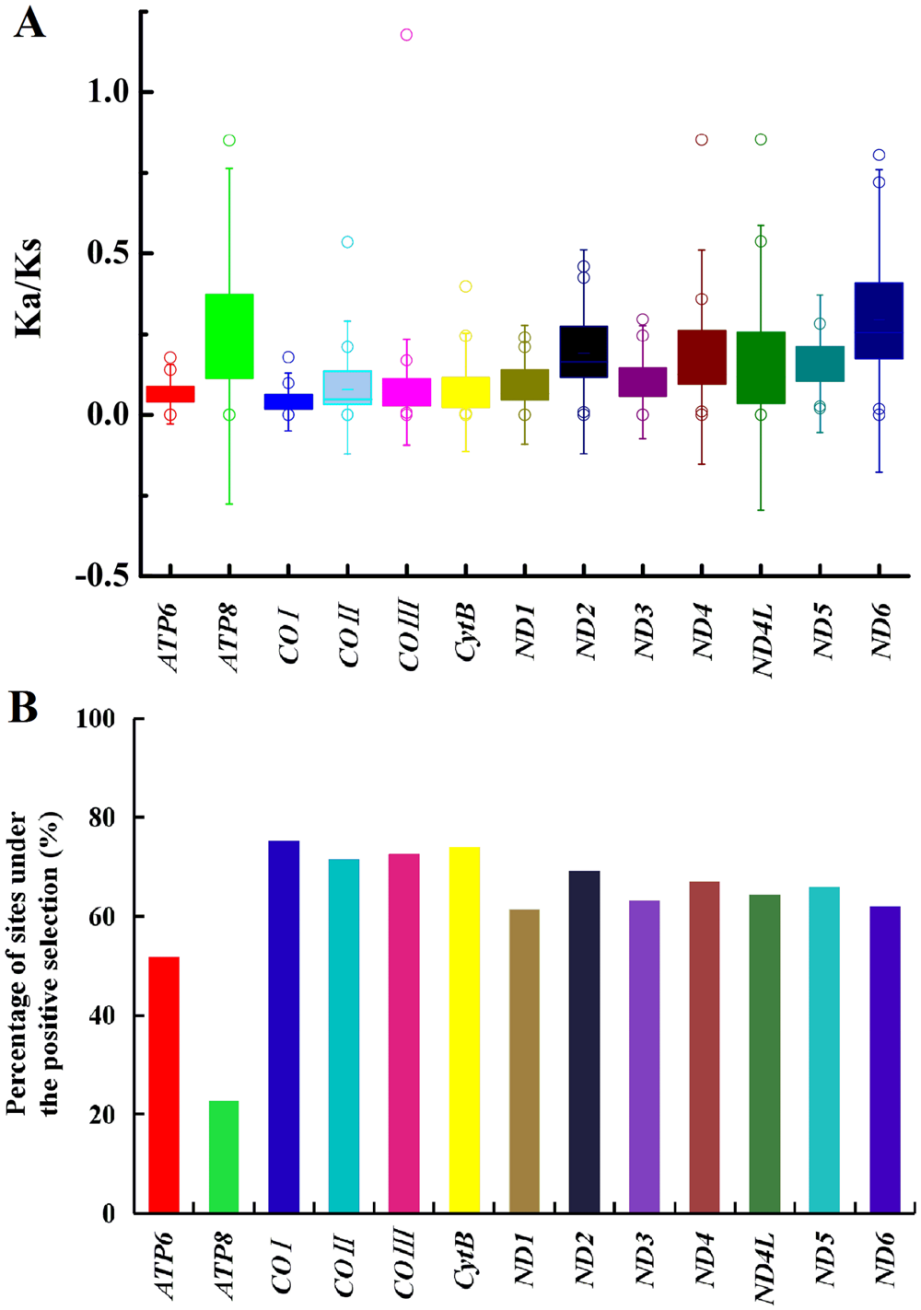
Positive selection estimated by FEL site-by-site analyses and pairwise divergence *Ka/Ks* ratio across mitochondrial protein-coding genes in 50 mosquito mitogenomes. (**A**) Ratios estimation. Box plot for pairwise divergence of *Ka/Ks* ratio for each one of the mitochondrial subunits. (**B**) Percentage of sites under positive selection.

To evaluate the selective pressures acting on mosquito mitogenomes, pairwise analyses of the non-synonymous (*Ka*) and synonymous (*Ks*) substitution ratio (*Ka/Ks*) were performed. The *Ka/Ks* ratio ranges from 0.039 ± 0.011 in *COI* to 0.295 ± 0.016 in *ND6*, and displays the following order: *COI* < *ATP6* = *COIII* < *CtyB* < *COII* < *ND1* < *ND3* < *ND4l* < *ND5* < *ND4* < *ND2* < *ATP8* < *ND6* (Fig. 5A). This result indicated that 13 PCGs in mosquito mitogenomes were globally evolving under negative constraints. Purifying selection seems to be particularly strong in Complex IV (*COI*, *COII* and *COII*) and Complex III (*CtyB*), in which *Ka/Ks* ratios are low and narrow (Fig. 5A). Indeed, due to their crucial roles in cellular respiration, PCGs have been expected to be mostly under purifying selection. Our results further support that *COI* is a suitable barcoding marker for mosquito phylogeny at some taxonomic levels. Subunits of NADPH complex I yielded higher average *Ka/Ks* values than other PCGs, which can be explained by relaxed purifying selection (here defined by *Ka/Ks* below one but significantly higher than other genes due to less conservative evolutionary constraints) or positive selection on them. The fixed effects likelihood (FEL) analyses (*P* < 0.05) revealed that the subunits with the highest percentage of codons under the negative selection presented as the following order: *COI* > *CytB* > *COIII* > *COII* > *ND2* > *ND4* > *ND5* > *ND4l* > *ND3* > *ND6* > *ND1* > *ATP6* > *ATP8* (Fig. 5B). Both analyses confirmed that purifying selection might be the major selection constraint for the maintenance of the complete mitogenome.

**Figure 6:**
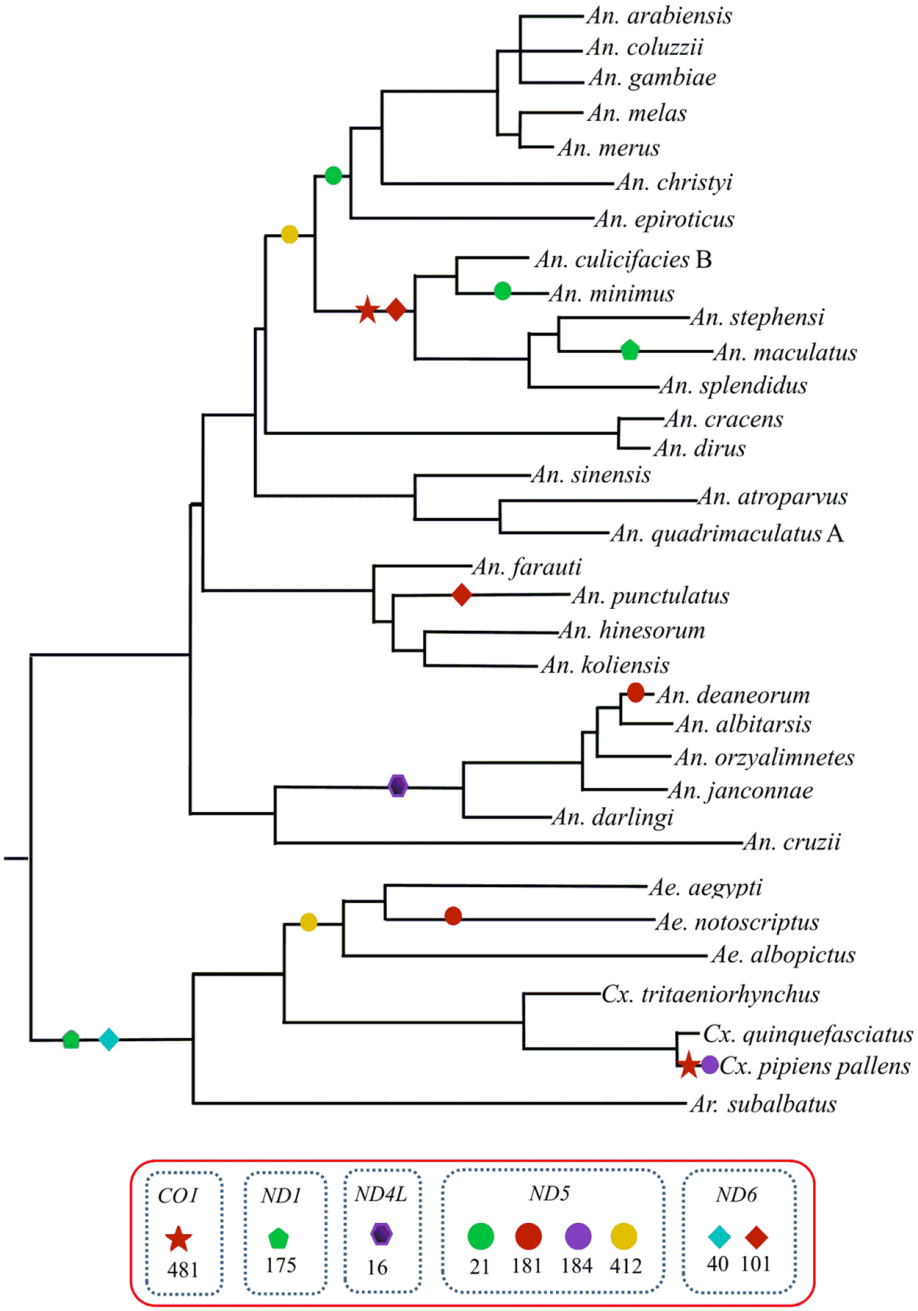
Adaptive evolution analyses based on MEME branch-site selection model. Identification of the maximum likelihood tree branches to test the adaptive evolution of each of the 13 PCGs in 34 representative mosquito species. The putatively positive selection sites with posterior probabilities ≥95% (p-value < 0.05) were marked with different symbols on the represented neighbor-joining tree of ATP6.

Given strong evolutionary constraints, it does not exclude the possibility for positive selection acting on single gene or few codon positions. From branch-site model test, the mixed effects model of evolution (MEME) analysis (posterior probability ≥ 95%) was able to identify a few positive selected codons in gene *ND1*, *ND4L*, *ND5*, *ND6* and *COX1* (Fig. 6). Therefore, changes in mitochondrial energy transduction system may play a crucial role in the evolution of mosquitoes. Since the mosquitoes occupy a variety of climates and habitants, the polymorphisms of some of OXPHO genes may facilitate climatic adaptions, long distance host-seeking and the host-parasites co-evolution. However, no positive selection acting on the protein-coding genes of four *Anopheles* mosquito mitogenomes was detected^46^. This result is largely due to few sequences available, very low sequence divergence, or lack of power of the analysis.

### Substitution saturation analysis

Plots of transversion and transition against the genetic distance showed a linear relationship for all datasets (Supplementary Fig. 4). The index of substitution saturation (*Iss* for PCG1 = 0.153, *Iss* for PCG2 = 0.071, and *Iss* for PCG3 = 0.176) was significantly lower (*P* = 0.000) than the critical value of the index of saturation (*Iss.c* for PCG1 = 0.831*, Iss.c* for PCG2 = 0.831 and *Iss.c* for PCG3 = 0.843), implying that the nucleotides of 50 mosquito mitogenomes experienced little substitution saturation, but they are qualified for the phylogenetic reconstruction.

### Phylogeny of 50 mosquito mitogenomes

**Figure 7:**
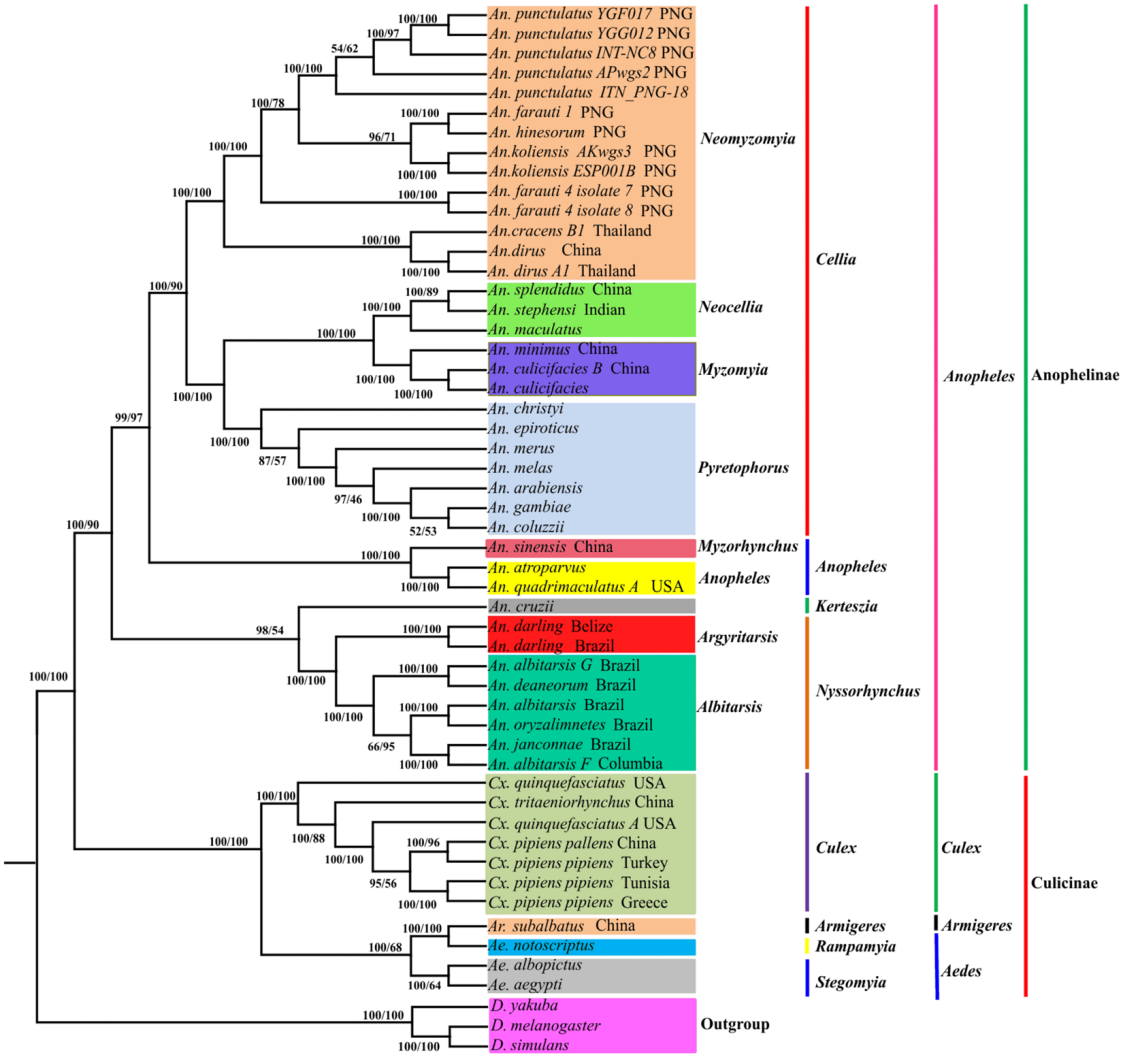
Reconstruction of phylogenetic tree determined by RAxML rapid bootstrap and Bayesian inference methods based on all sites (PCG123) of the concatenated protein-coding genes of 50 mosquito mitogenomes. Bayesian posterior probabilities (BPP) (left) and bootstrap support values (BP) are shown at relevant branches of the BI tree.

The phylogenetic analysis based on Bayesian inference (BI) and Maximum- likelihood (ML)-PCG123 produced an identical tree topology (Fig. 7). However, disparities between different datasets and inferring approaches were also evident (Supplementary Figs 5–8). The third codon position has proved to be less restricted by purifying selection and easily saturated with substitutions^47^, and therefore it was usually excluded in the phylogenetic analysis^48^. In the present study, however, the removing of the third codon position did not generate a better reliable tree topology as well as the concatenated amino acid sequence. In addition, the phylogenetic analyses based each individual gene also failed to provide identical tree topologies with these based on the concatenated 13 PCG123 (data not shown).

### Anophelinae Subfamily

The monophyly of Anophelinae subfamily containing four *Anopheles* subgenera (*Cellia*, *Anopheles*, *Keterszia* and *Nyssorhynchus*), and the sister relationships of two subgenera *Cellia* and *Anopheles* were recovered with a high statistical support, which is consistent with the traditional taxonomic views based on morphology and molecular phylogenetic studies^1, 5, 49^.

### Subgenus *Cellia*

Two major clades (*Neomyzomyia*, and *Pyretophorous* + (*Myzomyia* + *Neocellia*)) were clearly recognized in BI-tree and ML-PCG123 tree. As shown in other studies, *Neomyzomyia*, *Pyretophorous*, *Myzomyia* and *Neocellia* were shown to be monophyletic groups based on ribosomal (*18S RNA* and *28S RNA*) and individual mitochondrial genes (*COI* and *COII*)^5^. Contrary to our findings, the relationships within *Cellia* were poorly resolved by the morphological analysis^1^.

Phylogenetic analysis of morphological and molecular data indicated that the AP Group contains at least 13 sibling species^19^. However, their evolutionary relationships were not well resolved and sometimes were contradictory^20, 21^. A phylogenetic study using *18S RNA* and maximum parsimony inferring method recovered *An. koliensis* as the basal lineage of Farauti clade, and *An. farauti* 4 as the basal lineage of *Punctulatus* clade^50^. Logue *et al*.^51^ extended molecular characterizations by mitogenome analysis and concluded that *An. farauti* 4 was the most divergent, while *An. farauti.s.s* and *An. hinesorum* were most closely related to *An. punculatus*. The monophyly of the *An. punctulatus* Group was also recovered in BI-PCG123 and ML-PCG123 trees (Fig. 7) and formed the basal lineage of *An. farauti* 4, which further supported that AP group in PNG was colonized through a single migration event followed by speciation^51^. However, not all members of *An. farauti-*like species were clustered together because *An. koliensis* was consistently grouped with *An. farauti* and *An. hinesorum*.

Phylogenetic construction of *An. gambiae* Complex based on molecular markers was complicated, largely due to the degree of genetic similarity caused by the ancestral polymorphism and introgression. The sister relationship between *An. gambiae* and *An. arabiensis* was also confirmed in PCG123 data analysis. However, inversion phylogeny recovered *An. gambiae* and *An. merus* as sister taxa^52^. Our results revealed that *An. christyi* branched out earlier than *An. epiroticus* in BI- and ML-PG123, BI- and ML-AA (amino acid) trees, but in BI- and ML-PCG12 trees *An. epiroticus* branched out prior to *An. christyi* (Fig. 7; Supplementary Figs 5–8)

### Subgenera *Nyssorhynchus* and *Keterszia*

The monophyly and sister relationship of the subgenera *Nyssorhynchus* and *Keterszia* was in agreement with previous studies based on morphological or molecular data (Fig. 7; Supplementary Fig. 7). However, it was worthy to note that *Nyssorhynchus* was recovered as the sister to the clade including subgenera *Kerteszia* and *Stethomyia* based on mitochondrial gene *COI* and *COII*, and 5.8S rRNA^53^. In the present study, *An. cruzii* was recovered as the basal lineage of genus *Anopheles* in BI-PCG12 tree, ML- and BI-AA trees (Supplementary Figs 5, 6 and 8), suggesting that these datasets were less phylogenetically informative. Only one *Keterszia* species was included in the present study, more mitogenomic sequences will be helpful to resolve the monophyletic status of *Keterszia*. The sister relationship between subgenus *Cellia* and *Nyssorhynchus* + *Kerteszia* was recovered by morphological data analysis^47^, but this relationship was not supported in their recent study^1^. Our BI- and ML-PCG123 phylogenetic trees strongly supported the sister relationship of (*Nyssorhynchus* + *Kerteszia*) and (*Cellia* + *Anopheles*), which was consistent with the phylogeny inferred from 1,085 orthologs in 18 mosquito genomes^54^. The discrepancies between different analyses are likely due to the number and selection of samples, different molecular markers and computerized methods, which varied significantly among previous studies.

For the subgenus *Nyssorhynchus*, many approaches have been tried to resolve the phylogeny of Albitarsis Complex, including morphology, behavior, alloenzyme and molecular analysis^9, 18, 55–58^. However, the complete taxonomic picture for Albitarsis Complex is not yet clear, because there are conflicting tree topologies. The monophyly of Albitarsis Complex was previously recovered based on gene *ND6* and combined data sets^58^, ribosomal internal transcribed spacer 2 (ITS2) and rDNA^59^. However, this group was less well resolved in the *white* gene tree, which placed *An. marajoara* as a basal lineage to {*An. albitarsis* B (*An. albitarsis* [*An. deaneorum* 1, *An. deaneorum* 2])}^58^. *An. albitarsis* was recovered as a sister species to *An. deaneorum* in the *white* gene tree, but it was recovered as a sister to the remaining Albitarsis species in *ND6* gene tree. Another study based on the concatenated sequences of *COI* + *ND4* + *ITS2* + *D2* supported the relationship of *An. albitarsis* + [*An. albitarsis* B (formerly *An. oryzalimnetes*) + (*An. marajoara* + *An. deaneorum*)]^56^. Our BI- and ML-PCG123 trees supported the monophyly of Albitarsis Complex, but *An. albitarsis* G was recovered as a sister specie of *An. deaneorum*, and (*An. albitarsis* G + *An. deaneorum*) and formed the basal lineage of [(*An. albitarsis* + *An. albitarsis*) + (*An. Janconnae* (formerly *An. albitarsis* E) + *An. albitarsis* F].

### Culicinae Subfamily

Morphological and molecular evidence indicated that *Aedini* was a monophyletic taxon^60, 61^. However, *Aedini* was not recovered as a monophyletic group by Wilerson *et al*.^62^. The monophyly of *Aedini* was supported in BI- and ML-PCG123 trees, and *Ar. subalbatus* (*Armigeres* subgenus) was recovered as a sister of *Aedini* genus. Our results also found that Pipiens Complex was paraphyletic due to the inclusion of *Cx. quinquefasciatus* A USA.

### Divergence time estimation of mosquito species

**Figure 8:**
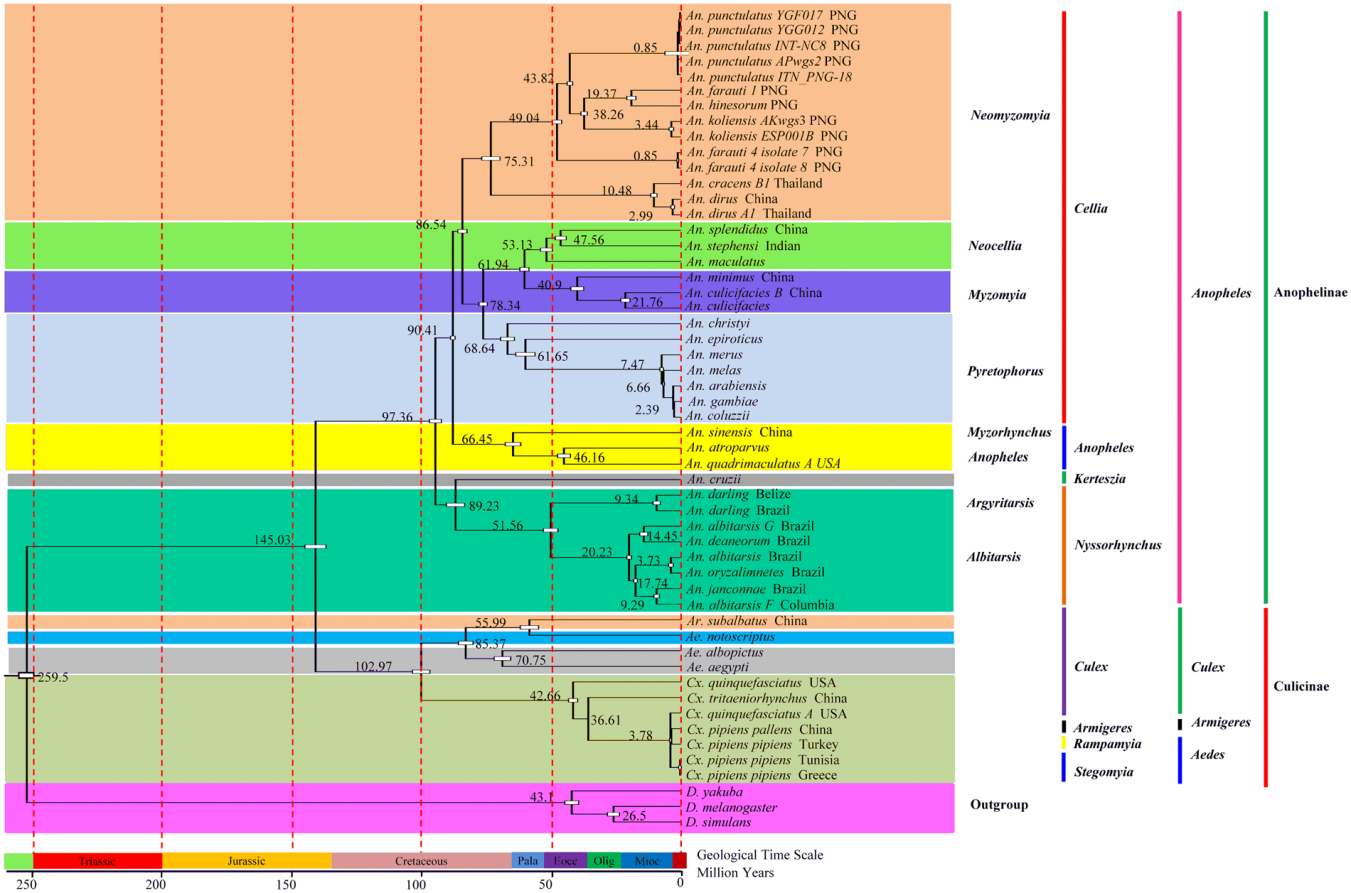
Evolutionary timescale for subfamily Anophelinae and Culicinae inferred from mtgenome PCG123 datasets based on BI tree. Numbers near the nodes indicate the average divergence time estimated (million years, Mya). In the geological time scale: Pala, Eoce, Olig and Mioc indicate Palaeocene, Eocene, Oligocene and Miocene, respectively.

Divergence time analysis based on PCG123 data revealed that the split date between subfamily Culicinae and Anophelinae was ~145.03 Mya, in the late Jurassic (Fig. 8). This estimation is in reasonable agreement with the report by Krzywinski *et al*.^63^, who speculated the divergence time between *Aedes* (*Stegomyia*) and *Anopheles* was ~146 Mya based on mitochondrial DNA sequences. However, Zhou *et al*.^64^ and Bertone *et al*.^65^ pushed backward the split date to ~122 Mya, and the time estimated by Moreno M *et al*.^46^ and Chen *et al*.^66^ was ~190 Mya (Early Jurassic) and 217.5 Mya (Late Triassic), respectively. The different evolutionary patterns of the molecular marker (nuclear DNA *vs* mitochondrial DNA), besides the algorithm and incomplete species sampling, partially explain the apparent incongruence between the estimations. Both Zhou *et al*.^64^ and Chen *et al*.^66^ estimated the divergence time using gene orthologous at genome level, whereas the study by Bertone *et al*.^65^ was aimed at deeper divergence within lower Diptera not within Culicidae and only a single molecular marker (28S rDNA) was used. Our estimation is also consistent with other evidences suggesting that mosquitoes likely originated in the Jurassic. The *Anopheles* radiation occurred during the early Cretaceous, and the split date between subgenera *Anopheles* and *Cellia* was estimated at ~90.41 Mya. This timescale inferred here is in agreement with the previous report ~90 Mya^12^, and ~93.6 Mya^46^. However, the last common ancestor of *Anopheles* was inferred in the Early Cretaceous (~113 Mya), and ~100 Mya based on 16 *Anophele*s genome^54^. *Kerteszia* and *Nyssorhynchus* are both distributed in South America, and was regarded as sister taxon. The divergence date of subgenera *Nyssorhynchus* was estimated at ~79 Mya^46^ or ~ 94 Mya^51^. Our estimation (~89.23 Mya) for *Nyssorhynchus* was comparable with those studies. Within the subgenus *Cellia*, the split date between *Myzomyia* (*An. minimus* + *An. culicifacies*) and *Neocellia* (*An. stephensis*) was ~61.94 Mya. The most recent ancestor of *An. dirus* from China and *An. dirus* from India was estimated at ~2.99 Mya.

## Materials and Methods

### Sample collection and DNA extraction

*An. stephensi* strain Hor (originally collected from India) and *An. dirus* (Hainan, China) were reared in the Third Military Medical University, China. Adult individuals were collected and stored at −80 °C. Total genomic DNA from a single adult was extracted using the modified sodium dodecyl-sulphate (SDS)/proteinase K method as described before^67^. The DNA was examined on 1% agarose gel and then used for PCR amplifications.

### PCR amplification and sequencing

To amplify the entire mitogenome of *An. stephensi* and *An. dirus*, 18 pairs of primers were designed based on the known mitogenomes of mosquito species (Supplementary Table 1), and PCR amplifications were conducted in 50 μl reaction mixture, including 5 μL of 25 mM MgCl_2_, 5 μL of 10 × PCR buffer (Mg^2+^ free), 8 μL of dNTP (2.5 mM each), 2 μL of each primer (10 mM), 2.5 U of Taq DNA polymerase (Takara, Japan) and 2 μL template DNA. The amplification conditions were as follows: an initial denaturation at 94 °C for 5 min, followed by 35 cycles of 94 °C for 1 min, 48–55 °C (depend on different primer pairs) for 45 s, and 68 °C for 1 min, and a final extension at 72 °C for 10 min. All PCR products were separated on 1% agarose gel by electrophoresis, purified using a QIAquick Gel Extraction Kit (Qiagen, China) and then sequenced. The control region was cloned into pMD-19T vector and then transformed into chemical competent *Escherichia coli* DH5α cells. The positive clone was sequenced at least three times.

### Mitogenome sequence assembly and analysis

Raw nucleotide sequences were trimmed to remove the low quality bases and assembled using DNAMAN V4.0 (Lynnon Biosoft). Annotations of the protein-coding genes, *rrnL* and *rrnS* gene were performed by a combination of BLAST searching and ORF Finder in GenBank, and MITOS^68^. Transfer RNA genes were identified using MITOS^68^, tRNAscan-SE^69^, and DOGMA^70^. The identification of tRNA^*Arg*^ and tRNA^*Ser*^, and the secondary structures of all tRNA genes were analyzed by comparison with the nucleotide sequences of other well-known mosquito tRNAs. Sequence motifs in the control region were identified using the Tandem Repeats Finder program^71^. AT- and GC-skew were calculated with the formula: AT skew = (A − T)/(A + T) and GC skew = (G − C)/(G + C)^72^. Codon usage bias was evaluated by calculating of effective number of codon (ENC) with the CodonW^73^. The relative synonymous codon usage (RSCU) was calculated by the DNAStar^74^, clustered and displayed using Cluster3.0 and Java TreeView1.23^75^, respectively.

### Tracking positive selection events on protein-coding genes

To detect evidence of selection on each protein-coding gene, amino acid sequence alignments were independently conducted by MAFFET program^76^. Nucleotide sequences were aligned based on the alignment of amino acid sequences to maintain the reading frame using the RevTrans^77^. The pair-wise comparison of the ratios of non-synonymous substitutions (*Ka*) and synonymous (*Ks*) substitutions was conducted using DnaSP5.0^78^. Further analyses were performed using the MEME (Mixed Effects Model of Evolution) method on Datamonkey server (www.datamonkey.org), which allows the rate of *Ka/Ks* to vary from site to site (fixed effect) or from branch to branch at a site (random effect). For the analysis, the best-fitting nucleotide substitution model for *ND2* (model 012034), *ND4* (model TrN93) and *ND6* (model 011120) were predicted on Datamonkey server.

### Sequence alignment, saturation and phylogenetic analyses

Two complete mitogenome sequences obtained in the present study, and 48 complete or nearly complete mosquito mitogenome sequences from NCBI or our unpublished data were used for phylogenetic analyses (Supplementary Table 2). Sequence data from three *Drosophila* species (*D*. *melanogaster* NC_001709, *D. yakuba* NC_001322, and *D. simulans* NC_005781) were included as the outgroup. Nucleotide and amino acid sequences of 13 protein-coding genes were separately aligned using MAFFT^76^. After removing the poorly aligned and divergent regions using Gblocks^79^, the individual alignment was then concatenated following their orders in the mitogenome with Sequence Matrix v1.7.6^80^. Substitution saturation was tested by plotting the number of transitions and transversions against genetic divergence (GTR) using DAMBE^81^.

For phylogenetic analyses, three data types were used: 1) concatenated amino acid sequences of 13 protein coding genes; 2) all three nucleotides in codons of 13 protein-coding genes (PCG123 with 11192 nucleotides); 3) the 1^st^ and 2^nd^ nucleotide in codons of 13 protein-coding genes (PCG12 with 7462 nucleotides). The General Time Reversible (GTR) model incorporating invariant sites and a gamma distribution (GTR + I + G) for nucleotide datasets was selected by Modeltest^82^ using the Akaike Information Criterion (AIC), and the site-heterogeneous model GTR + CAT (CAT model, named due to classifying sites into categories) was used for amino acid sequences. Maximum likelihood analyses were conducted under the best model using RAxML^83^. Node support was estimated by analyzing 1000 bootstrap replicates. Bayesian inference was conducted using MrBayes^84^ and two independent runs with four chains (three heat and one cold) were performed simultaneously for 1,000,000 generations. The runs were stopped till the average deviation of split frequencies fall below 0.01. Stationarity is considered to be reached when estimated samples size (ESS) value is above 100 and potential scale reduction factor (PSRF) approach 1.0. The chains were sampled every 100 generations with the discard of the first 25% as burn-in. Posterior probabilities (PP) were calculated in a consensus tree. The topology of the best-scoring trees were visualized and edited in FigTree V1.4 (http://www.molecularevolution.org).

### Divergence time Estimation

Estimation of divergence times among mosquitoes were calculated in BEAST1.8.3^85^. The GTR + I + G substitution model, empirical base frequencies and speciation Yule model were applied as Tree prior. Two independent MCMC runs were each performed for a total of 1,000,000 generations with the first 25% burned-in under the uncorrelated lognormal relaxed clock and sampled every 1000 generations to estimate the divergence time. The split date (259.9 Mya) between *Anopheles* and *Drosophila* was used as the calibration^63, 86^.

## Electronic supplementary material

Supplementary Information
