# Supplementary material for: Complete mitochondrial genomes of Anopheles stephensi and An. dirus and comparative evolutionary mitochondriomics of 50 mosquitoes
Source: Sci Rep. 2017 Aug 9;7:7666. doi: 10.1038/s41598-017-07977-0 (PMC5550476; doi:10.1038/s41598-017-07977-0)
Supplement: Supplementary file 1 — Supplementary Information [file 41598_2017_7977_MOESM1_ESM.pdf]

Additional Information For

Complete mitochondrial genomes of *Anopheles stephensi* and *An. dirus* and comparative evolutionary mitochondriomics of 50 mosquitoes

You-Jin Hao, Yi-Lin Zou, Yi-Ran Ding, Wen-Yue Xu, Zhen-Tian Yan, Xu-Dong Li, Wen-Bo Fu, Ting-Jing Li & Bin Chen

Supplementary Table 1. Sequences and annealing positions of the primers used for the amplification of the mtDNA fragments in *Anopheles stephensi* and *An. dirus.*

Supplementary Table 2. List of mosquito species and the information of their mitogenomes included in the present study.

Supplementary Table 3. A+T content, AT- and GC-skew in 50 mosquito mitogenomes.

Supplementary Table 4. Pairwise-genetic difference of orthologous *tRNAs* in 39 mosquito mitogenomes

Supplementary Figure 1. ENC-GC3 plot of 13 individual protein-coding genes in 50 mosquito mitogenomes. The dashed line (standard curve) indicates the expected ENC. Points on or close the standard curve means codon bias caused by mutation pressure. Otherwise, bias is affected by natural selections or other factors.

Supplementary Figure 2. Hierarchal clustering analysis of the relative synonymous codon usage for all 13 PCGs of 50 mosquito mitogenomes. Each square on the self-organizing map represents the RSCU value of a codon (shown in columns) corresponding to the species (shown in rows). Deeper red indicates more usage of a codon and bluer color indicates rarer usage of codon in the corresponding gene.

Supplementary Figure 3. Secondary structure of tRNA families in 38 mosquito mitogenomes. The nucleotide substitution pattern for each tRNA family was modeled using the ancestor insect tRNA as the structural reference. tRNAs with

red names are localized on the minority strand.

Supplementary Figure 4. Substitution saturation of 13 concatenated protein-coding genes (PCGs). Transitions (red) and transversions (green) were plotted against the GTR distance. A) First codon position of 13 PCGs; B) Second codon position of 13 PCGs; C) Third codon position of 13 PCGs; D) All sites of 13 PCGs.

Supplementary Figure 5. Phylogenetic tree deduced by Bayesian-inference method based on the 1st and 2nd nucleotide of codons of the concatenated 13 PCGs of 50 mosquito mitogenomes. Bayesian posterior probabilities are shown for the corresponding branches. Branches in red indicate the different placing from the phylogenetic relationship inferred from PCG123.

Supplementary Figure 6. Reconstruction of phylogenetic tree inferred by Bayesian inference method based on the concatenated 13 proteins of 50 mosquito mitogenomes. Bayesian posterior probabilities (BPP) are shown at relevant branches of the BI tree.

Supplementary Figure 7. Maximum-likelihood phylogenetic tree inferred by RAxML based on the 1st and 2nd nucleotide of codons (PCG12) of the concatenated 13 PCGs of 50 mosquito mitogenomes. Larger than 50% bootstrap support values from 1000 replications are shown on the corresponding branches. Branches in red indicate the different placing in the phylogenetic trees deduced from PCG12 and PCG123.

Supplementary Figure 8. Maximum-likelihood phylogenetic tree inferred by RAxML method based on of the concatenated 13 proteins of 50 mosquito mitogenomes. Bootstrap support values (BP) over 50% are shown on the corresponding branches. Branches in red indicate the different placing from phylogenetic relationship inferred from PCG123.

Supplementary Table 1. Sequences and annealing positions of the primers used for the amplification of the mtDNA fragments in *Anopheles stephensi* and *An. dirus.*

Primer Name	Sequence 5'-3'
1-F147	AATTAAGCTATTGGGTTCATACCC
1-R1418	GGCTGAATTTTAGGCGATAAATTGTAAA
2-F1237	GTTAAATAAACTAATAGCCTTCAAA
2-R2355	GCTCGTGTATCTACGTCTATTCC
3-F2162	CCTGGATTTGGAATAATTTCT
3-R2983	TGCACTAATCTGCCATATTAGA
4-F2961	TCTAATATGGCAGATTAGTGCA
4-R4092	GAGAATAAGTTTGTTATCATTTTCA
5-F3748	CATTAGATGACTGAAAGCAAGTA
5-R4948	CGAGTTACATCTCGTCATCATTG
6-F4438	TACCTTTTATGGTATGCATTGAA
6-R5411	GCTGCTTCAAATCCAAAATGATG
7-F5170	TTCACAAGCTACTCAAGGATT
7-R6425	TTTTGGAGGACAAATATTA
8-F6254	ATCTCCCTAACATCTTCAGTG
8-R7249	ATTTATGGCGGGGTTGGGTGC
9-F7152	CTCAAAATTAGCCCCTAATCCAG
9-R8315	TTCTGGGAATATGGCAGCTC
10-F8158	TCATATCATTGACACCACA
10-R9156	GAGGGTATCAACCTGAACG
11-F8806	TTATAGAACCAGAAACAGG
11-R9789	TTAGTTTACAAGACTAATG
12-F9527	ACTCCTTCACATACACAAAAAGT
12-R10727	CATAGTAAACACCACGACCTA
13-F10464	AGTAGATTTACCTGCACCTTCAAA
13-R11452	TGGATCTTCTACAGGTCGAGCTC
14-F10807	GTTCTACCTTGAGGACAAATATC
14-R12155	GTAGCCCAAACCATTTCTTATGA
15-F11741	CGAGGTAAAGTTCCACGAACTCA
15-R12906	TTACCTTAGGGATAACAGCGTAA
16-F12754	CCGGTCTGAACTCAGATCATGTA
16-R14124	ATATGCACACATCGCCCGTC
17-F13764	TTTGATAAACCCTGATACACAAG
17-R14662	GTGCCAGCAGTCGCGGTTATAC
18-F14478	ATAATAGGGTATCTAATCCTAGT
18-R183	ACCTTTATAAGTGGGGTATGAACC

Supplementary Table 2. List of mosquito species and the information of their mitogenomes included in the present study.

Species with taxonomic taxa information	Accession No.	Size (bp)	Collecting locations	References
**Culicinae Subfamily**				
**Aedini Tribe**				
***Aedes* Genus**				
***Rampamyia* Subgenus**				
Ae. notoscriptus	NC025473	15846	Unknown	Direct Submission
***Stegomyia* Subgenus**				
**Aegypti Group**				
Ae. aegypti	NC010241	16655	Unknown	Behura, *et al.*[1]
**W-albus Group**				
Ae. albopictus	NC006817	16665	Taiwan	Direct Submission
***Armigeres* Genus**				
***Armigeres* Subgenus**				
Ar. subalbatus	n/a	14719	China	Unpublished
**Culicini Tribe**				
***Culex* Genus**				
***Culex* Subgenus**				
**Pipiens Group**				
**Pipiens Complex**				
Cx. pipiens pallens	KT851543	14856	China	Luo, *et al.*[2]
Cx. quinquefasciatus A	HQ724617	14856	USA	Direct Submission
Cx. pipiens pipiens	HQ724614	14856	Tunisia	Direct Submission
Cx. pipiens pipiens	HQ724615	14856	Greece	Direct Submission
Cx. quinquefasciatus	NC014574	15587	USA	Behura, *et al.*[1]
Cx. pipiens pipiens	HQ724616	14856	Turkey	Direct Submission
**Sitiens Group**				
**Vishnui Subgroup**				
Cx. tritaeniorhynchus	KT851544	14844	China	Luo, *et al.*[2]
**Anophelinae Subfamily**				
***Anopheles* Genus**				
***Nyssorhynchus* Subgenus**				
**Argyritarsis Section**				
**Albitarsis Series**				
**Albitarsis Group**				
**Albitarsis Complex**				
An. albitarsis F	HQ335349	15418	Columbia	Krzywinski, *et*
An. albitarsis G	HQ335346	15474	Brazil	Krzywinski, *et*
An. albitarsis	NC020662	15413	Brazil	Krzywinski, *et*
An. deaneorum	NC020663	15424	Brazil	Krzywinski, *et*
An. oryzalimnetes	HQ335345	15422	Brazil	Krzywinski, *et*
An. janconnae	HQ335348	15425	Brazil	Krzywinski, *et*
**Argyritarsis Series**				
**Darlingi Group**				
An. darling Beliz	GQ918272	15386	Belize	Moreno, *et al.*[4]

An. darling Brazil	GQ918273	15385	Brazil	Moreno, *et al.*[4]
***Cellia* Subgenus**				
**Myzomyia Series**				
**Funestus Group**				
**Minimus Subgroup**				
**Minimus Complex**				
An. minimus	KT895423	15395	China	Hua, *et al.*[5]
**Culicifacies Subgroup**				
An. culicifacies B	NC027502	15330	China	Hua, *et al.*[5]
An. culicifacies	NC028216	15364	Unknown	Direct Submission
**Neocellia Series**				
An. stephensi	KT899888	15371	Indian	This study
**Jamesii Group**				
An. splendidus	KX887321	15363	China	Unpublished
**Maculatus Group**				
An. maculatus	NC028218	14850	Unknown	Direct Submission
**Neomyzomyia Series**				
**Punctulatus Group**				
An. punctulatus ITN_NC-8	JX219737	15198	Papua New Guinea	Logue, *et al.*[6]
An. punctulatus ITN_PNG-18	JX219738	15200	Papua New Guinea	Logue, *et al.*[6]
An. punctulatus YGF017	JX219739	15085	Papua New Guinea	Logue, *et al.*[6]
An. punctulatus YGG012	JX219740	14965	Papua New Guinea	Logue, *et al.*[6]
An. punctulatus APwgs2	JX219744	15045	Papua New Guinea	Logue, *et al.*[6]
An. koliensis AKwgs3	JX219742	15412	Papua New Guinea	Logue, *et al.*[6]
An. koliensis ESP001B	JX219743	15412	Papua New Guinea	Logue, *et al.*[6]
**Farauti Complex**				
An. farauti 1	JX219741	15412	Papua New Guinea	Logue, *et al.*[6]
An. farauti 4 isolate 7	JX219735	15412	Papua New Guinea	Logue, *et al.*[6]
An. farauti 4 isolate 8	JX219736	15412	Papua New Guinea	Logue, *et al.*[6]
An. hinesorum	NC020769	15336	Papua New Guinea	Logue, *et al.*[6]
**Leucosphyrus Group**				
**Leucosphyrus Subgroup**				
**Dirus Complex**				
An. cracens B1	NC020768	15412	Thailand	Logue, *et al.*[6]
An. dirus A1	JX219731	15404	Thailand	Logue, *et al.*[6]
An. dirus	KT899887	15406	China	This study
**Pyretophorus Series**				
An. christyi	NC028214	14967	Unknown	Direct Submission
**Sundaicus Complex**				
An. epiroticus	NC028217	15379	Unknown	Direct Submission
**Gambiae Complex**				
An. arabiensis	NC028212	15369	Unknown	Direct Submission
An. coluzzii	NC028215	15441	Unknown	Direct Submission
An. gambiae	NC002084	15363	Unknown	Beard, *et al.*[7]
An. melas	NC028219	15366	Unknown	Direct Submission
An. merus	NC028220	15365	Unknown	Direct Submission
***Anopheles* Subgenus**				
An. atroparvus	NC028213	15458	Unknown	Direct Submission
An. quadrimaculatus A	NC000875	15455	USA	Mitchell, *et al.*[8]

An. sinensis	n/a	11816	China	Unpublished
Kerteszia **Subgenus**				
An. cruzii	NC024740	15449	USA	Direct Submission
**Drosophilidae (as outgroup)**				
Dr. melanogaster	NC_00170	19517	USA	Direct Submission
Dr. yakuba	NC_00132	16019	USA	Clary, *et al.*[9]
Dr. simulans	NC_00578	14972	USA	Direct Submission

References

1. Behura, S. K., Lobo, N. F., Haas, B., Debruyn, B., Lovin, D. D., Shumway, M. F. *et al*. Complete sequences of mitochondria genomes of *Aedes aegypti* and *Culex quinquefasciatus* and comparative analysis of mitochondrial DNA fragments inserted in the nuclear genomes. *Insect Biochemistry & Molecular Biology* **41**, 770-777 (2011).
2. Luo, Q. C., Hao, Y. J., Meng, F., Li, T. J., Ding, Y. R., Hua, Y. Q. *et al*. The mitochondrial genomes of *Culex tritaeniorhynchus* and *Culex pipiens pallens* (Diptera: Culicidae) and comparison analysis with two other Culex species. *Parasites & vectors* **9** (2016).
3. Krzywinski, J., Li, C., Morris, M., Conn, J. E., Lima, J. B., Povoa, M. M. *et al*. Analysis of the evolutionary forces shaping mitochondrial genomes of a neotropical malaria vector complex. *Molecular Phylogenetics & Evolution* **58**, 469-477 (2011).
4. Moreno, M., Marinotti, O., Krzywinski, J., Tadei, W. P., James, A. A., Achee, N. L. *et al*. Complete mtDNA genomes of *Anopheles darlingi* and an approach to anopheline divergence time. *Malar Journal* **9**, 127, doi:10.1186/1475-2875-9-127 (2010).
5. Hua, Y. Q., Yan, Z. T., Fu, W. B., He, Q. Y., Zhou, Y. & Chen, B. Sequencing and analysis of the complete mitochondrial genome in *Anopheles culicifacies* species B (Diptera: Culicidae). *Mitochondrial DNA*, **4**, 1-2 (2016).
6. Logue, K., Chan, E. R., Phipps, T., Small, S. T., Reimer, L., Henry-Halldin, C. *et al*. Mitochondrial genome sequences reveal deep divergences among *Anopheles punctulatus* sibling species in Papua New Guinea. *Malar Journal* **12**, 64, doi:10.1186/1475-2875-12-64 (2013).
7. Beard, C. B., Hamm, D. M. & Collins, F. H. The mitochondrial genome of the mosquito *Anopheles gambiae*: DNA sequence, genome organization, and comparisons with mitochondrial sequences of other insects. *Insect Molecular Biology* **2**, 103-124 (1993).
8. Mitchell, S. E., Cockburn, A. F. & Seawright, J. A. The mitochondrial genome of

Anopheles quadrimaculatus species A: complete nucleotide sequence and gene organization. *Genome* **36**, 1058-1073 (1993).

9. Clary, D. O. & Wolstenholme, D. R. The mitochondrial DNA molecular of *Drosophila yakuba*: nucleotide sequence, gene organization, and genetic code. *Journal of Molecular Evolution* **22**, 252-271 (1985).

Supplementary Table 3. A+T content, AT- and GC-skew in 50 mosquito mitogenomes.

Species	Genome without CR			PCGs		tRNAs		16sRNA		12sRNA		CR	
	AT (%)	AT skew	GC skew	Size (bp)	AT (%)	Size (bp)	AT (%)	Size (bp)	AT (%)	Size (bp)	AT (%)	Size (bp)	AT (%)
**Culicinae Subfamily**													
**Aedeni Tribe**													
***Aedes* Genus**													
***Rampamyia* Subgenus**													
Ae. notoscriptus	78.7	0.010	-0.173	11208	77.3	1490	79.9	1335	83.5	789	82.3	940	94.0
***Stegomyia* Subgenus**													
**Aegypti Group**													
Ae. aegypti	77.3	0.021	-0.201	11191	75.9	1488	79.3	1335	83.3	790	81.5	1709	93.6
**W-albus Group**													
Ae. albopictus	78.1	0.013	-0.173	11225	77.1	1483	79.4	1333	82.8	804	81.3	1772	91.6
***Armigeres* Genus**													
***Armigeres* Subgenus**													
Ar. subalbatus	78.7	0.01	-0.20	11153	77.7	1487	80.9	1307	83.3	452	77.9	n/a	n/a
**Culicini Tribe**													
***Culex* Genus**													
***Culex* Subgenus**													
**Pipiens Group**													
**Pipiens Complex**													
Cx. pipiens pallens	77.5	0.01	-0.15	11234	76.6	1482	79.0	1334	83.2	804	81.2	747	88.7
Cx. quinque fasciatus A	77.7	0.01	-0.16	11220	76.3	1483	78.8	1333	83.1	804	80.8	704	88.5

Cx. pipiens pipiens	77.6	0.01	-0.16	11216	76.5	1479	79.0	1333	83.2	785	80.6	n/a	n/a
Cx. pipiens pipiens	77.6	0.01	-0.16	11168	76.5	1475	79.0	1333	83.2	785	80.6	n/a	n/a
Cx. quinque fasciatus	78.0	0.01	-0.18	11220	76.3	1483	78.8	1333	83.1	804	80.8	704	88.5
Cx. pipiens pipiens	77.7	0.01	-0.16	11168	76.6	1475	78.9	1333	83.3	785	80.8	n/a	n/a
**Sitiens Group**													
**Vishnui Subgroup**													
Cx. tritaeniorhynchus	77.4	0.01	-0.16	11225	76.5	1490	78.8	1338	83.3	787	80.7	n/a	n/a
**Anophelinae Subfamily**													
***Anopheles* Genus**													
***Nyssorhynchus* Subgenus**													
**Arggritarsis Section**													
**Albitarsis Series**													
**Albitarsis Group**													
**Albitarsis Complex**													
An. albitarsis F	77.3	0.031	-0.162	11216	76.3	1478	78.6	1328	82.5	793	80.2	578	92.9
An. albitarsis G	77.0	0.031	-0.162	11216	76.0	1476	78.8	1332	82.4	793	80.5	615	92.5
An. albitarsis	77.1	0.031	-0.162	11216	76.0	1476	78.7	1326	82.1	793	79.8	575	93.2
An. deaneorum	77.2	0.03	-0.17	11216	76.2	1475	78.3	1328	82.2	793	80.0	581	92.3
An. oryzalimnetes	77.2	0.03	-0.16	11216	76.1	1477	78.7	1327	82.1	793	80.0	581	94.2
An. janconnae	77.1	0.03	-0.16	11216	76.0	1478	78.4	1327	82.5	793	79.7	575	92.4
**Arggritarsis Series**													
**Darlingi Group**													
An. darling Belize	77.6	0.03	-0.14	11196	76.8	1489	78.5	1394	82.6	793	80.0	554	93.7
An. darling Brazil	77.6	0.03	-0.14	11208	76.7	1489	78.4	1394	82.5	793	79.7	n/a	n/a
***Cellia* Subgenus**													

**Myzomyia Series**													
**Funestus Group**													
**Minimus Subgroup**													
**Minimus Complex**													
An. minimus	78.6	0.026	-0.141	11230	79.1	1509	79.0	1325	83.3	794	80.6	537	93.0
Culicifacies **Subgroup**													
An. culicifacies B	78.0	0.033	-0.151	11196	77.1	1523	78.5	1322	83.3	791	80.2	498	92.6
An. culicifacies	77.5	0.04	-0.16	11199	76.4	1474	78.6	1324	82.8	792	80.3	535	93.1
**Neocellia Series**													
An. stephensi	77.5	0.04	-0.14	11227	76.7	1476	79.1	1327	83.0	795	81.0	531	94.2
**Jamesii Group**													
An. splendidus	77.9	0.03	-0.16	11103	76.2	792	80.2	1338	80.7	792	80.2	519	93.2
**Maculatus Group**													
An. maculatus	77.6	0.04	-0.15	11196	76.5	1490	78.7	1325	83.2	796	80.4	n/a	n/a
**Neomyzomyia Series**													
**Punctulatus Group**													
An. punctulatus ITN_NC-8	78.2	0.04	-0.14	11196	77.1	1476	79.0	1326	83.1	797	80.6	n/a	n/a
An. punctulatus ITN_PNG-18	78.2	0.04	-0.14	11192	77.2	1476	79.0	1326	83.1	796	80.5	580	92.7
An. punctulatus YGF017	78.2	0.04	-0.14	11192	77.2	1476	79.0	1326	83.1	797	80.6	n/a	n/a
An. punctulatus YGG012	78.2	0.04	-0.13	11192	77.2	1476	79.0	1326	83.1	796	80.5	n/a	n/a
An. punctulatus APwgs2	78.2	0.04	-0.13	11192	77.2	1476	79.0	1326	83.0	796	80.5	n/a	n/a
An. koliensis AKwgs3	77.5	0.04	-0.16	11224	76.5	1478	78.5	1327	82.7	795	80.1	582	91.9
An. koliensis ESP001B	77.5	0.04	-0.16	11224	76.5	1478	78.5	1327	82.7	795	80.1	582	91.9
**Farauti Complex**													
An. farauti 1	77.8	0.04	-0.15	11224	76.8	1477	78.7	1325	82.7	798	80.5	580	92.0

An. farauti 4 isolate7	77.4	0.03	-0.15	11224	76.3	1477	78.9	1327	82.4	798	80.8	576	93.5
An. farauti 4 isolate8	77.4	0.03	-0.15	11224	76.3	1477	78.9	1327	82.4	798	80.8	576	93.5
An. hinesorum	77.5	0.04	-0.15	11224	76.4	1478	78.8	1325	82.9	798	80.6	505	92.5
**Leucosphyrus Group**													
**Leucosphyrus Subgroup**													
**Dirus Complex**													
An. cracens B1	77.4	0.029	-0.156	11224	76.3	1482	79.0	1325	82.8	798	81.0	572	93.2
An. dirus A1	77.6	0.03	-0.16	11224	76.7	1479	78.7	1325	82.7	798	80.8	566	92.3
An. dirus	77.5	0.03	-0.15	11224	76.7	1478	78.8	1326	82.4	798	80.8	568	92.3
**Pyretophorus Series**													
An. christyi	76.7	0.044	-0.173	11193	75.3	1477	78.3	1325	82.2	799	80.1	n/a	n/a
**Sundaicus Complex**													
An. epiroticus	77.2	0.03	-0.14	11196	76.1	1479	78.4	1325	82.3	798	80.1	535	91.8
**Gambiae Complex**													
An. arabiensis	77.0	0.035	-0.154	11199	76.0	1477	78.1	1325	82.5	800	79.5	530	94.5
An. coluzzii	77.0	-0.036	-0.153	11199	76.0	1478	78.1	1325	82.5	800	79.6	n/a	n/a
An. gambiae	77.0	0.03	-0.15	11230	76.0	1478	77.9	1325	82.5	800	79.6	519	94.2
An. melas	77.0	0.04	-0.16	11199	76.0	1477	78.1	1325	82.4	800	79.5	526	93.9
An. merus	77.0	0.04	-0.16	11199	75.9	1478	78.2	1325	82.5	800	79.8	525	94.3
***Anopheles* Subgenus**													
An. atroparvus	76.7	0.036	-0.147	11187	75.6	1486	78.5	1319	81.8	793	80.1	614	92.7
An. quadrimaculatus A	77.4	0.04	-0.18	11220	75.5	1474	78.6	1321	82.2	794	80.5	625	93.4
An. sinensis	78.4	0.03	-0.16	11231	77.1	1476	78.5	1328	82.4	797	79.9	586	92.6
***Kerteszia* Subgenus**													
An. cruzii	78.4	0.018	-0.165	11230	76.7	1475	79.4	1323	83.7	793	81.1	600	93.0

Supplementary Table 4. Pairwise-genetic difference of orthologous *tRNAs* in 39 mosquito genomes.

tRNA in genome order	BDps	pDis	MLdis
tRNA^{I}	3.7	0.055±0,022	0.061±0.025
tRNA^{Q}	**7.1**	**0.093±0.08**	**0.139±0.119**
tRNA^{M}	1.6	0.023±0.012	0.024±0.013
tRNA^{W}	1.8	0.027±0.015	0.03±0.016
tRNA^{C}	**7.7**	**0.114±0.104**	**0.179±0.134**
tRNA^{Y}	**8.1**	**0.119±0.1**	**0.192±0.134**
tRNA^{L1}	2.1	0.027±0.011	0.036±0.011
tRNA^{K}	2.0	0.033±0.028	0.037±0.033
tRNA^{D}	4.8	0.071±0.038	0.083±0.015
tRNA^{G}	2.6	0.038±0.014	0.041±0.016
tRNA^{R}	1.6	0.025±0.09	0.026±0.009
tRNA^{A}	2.3	0.036±0.019	0.039±0.022
tRNA^{N}	3.0	0.047±0.017	0.051±0.02
tRNA^{S2}	**3.4**	**0.055±0.025**	**0.061±0.031**
tRNA^{E}	2.1	0.03±0.014	0.035±0.015
tRNA^{F}	**7.8**	**0.11±0.103**	**0.183±0.15**
tRNA^{H}	**2.4**	**0.031±0.018**	**0.024±0.015**
tRNA^{T}	5.0	0.079±0.018	0.088±0.051
tRNA^{P}	**9.1**	**0.135±0.116**	**0.257±0.173**
tRNA^{S1}	2.2	0.03±0.014	0.032±0.015
tRNA^{L2}	**9.6**	**0.13±0.083**	**0.18±0.124**
tRNA^{V}	**0.95**	**0.009±0.003**	**0.007±0.001**

[1]BDps: base difference per sequence;

[2]MLdis: Maximum composite likelihood distance calculated for every pairwise-comparison of orthologous *tRNAs*;

[3]pDis: p-Distance calculated for every pairwise-comparison;

[4] tRNAs on the minority strand were marked in bold.

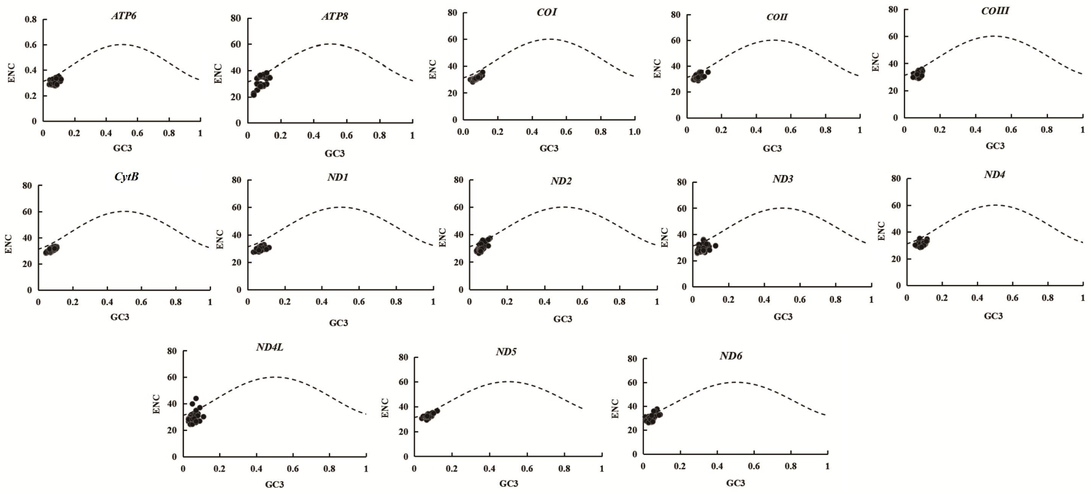

Supplementary Figure 1. ENC-GC3 plot of 13 individual protein-coding genes in 50 mosquito mitogenomes. The dashed line (standard curve) indicates the expected ENC. Points on or close the standard curve means codon bias caused by mutation pressure. Otherwise, bias is affected by natural selections or other factors.

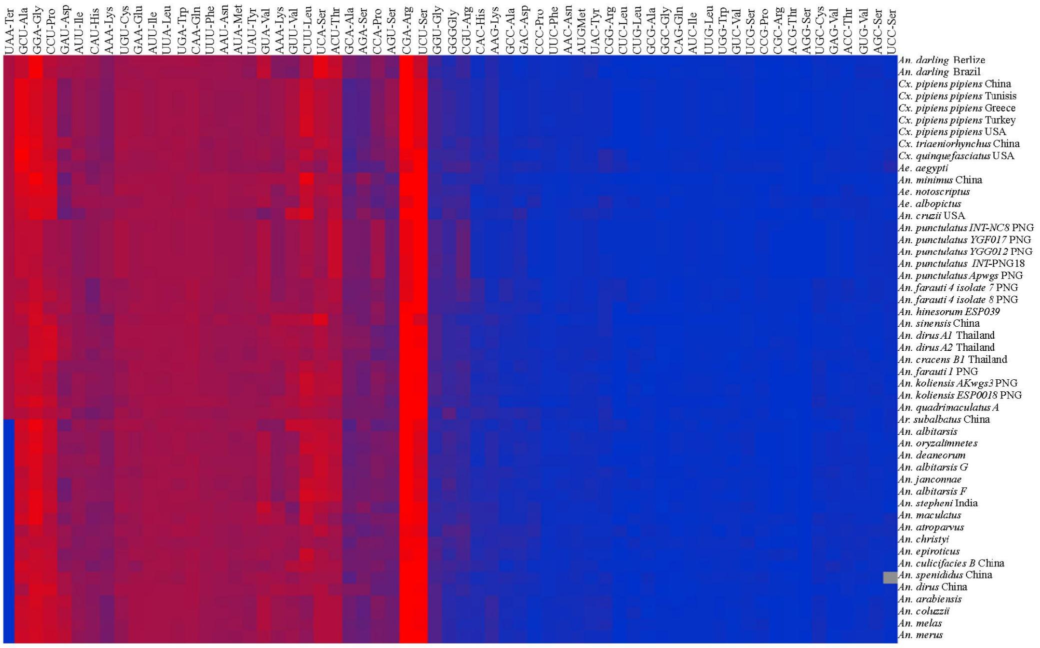

Supplementary Figure 2. Hierarchal clustering analysis of the relative synonymous codon usage for all 13 PCGs of 50 mosquito mitogenomes. Each square on the self-organizing map represents the RSCU value of a codon (shown in columns) corresponding to the species (shown in rows). Deeper red indicates more usage of a codon and bluer color indicates rarer usage of codon in the corresponding gene.

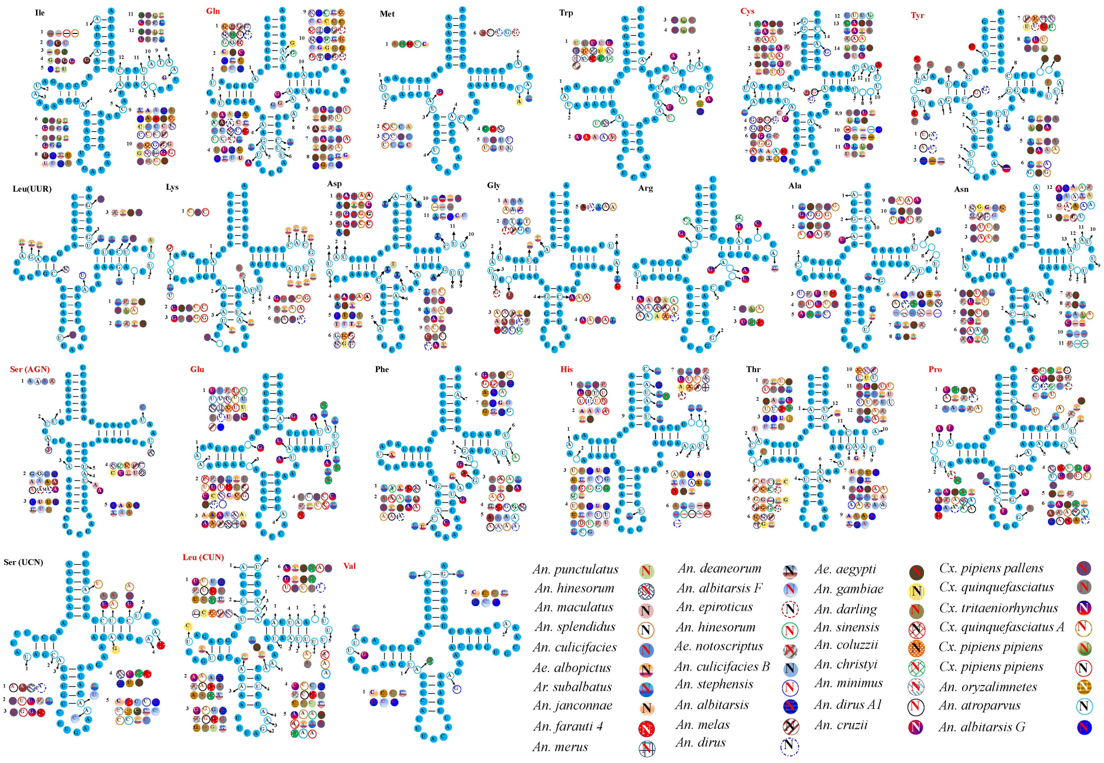

Supplementary Figure 3. Secondary structure of tRNA families in 38 mosquito mitogenomes. The nucleotide substitution pattern for each tRNA family was modeled using the ancestor insect tRNA as the structural reference. tRNAs with red names are localized on the minority strand.

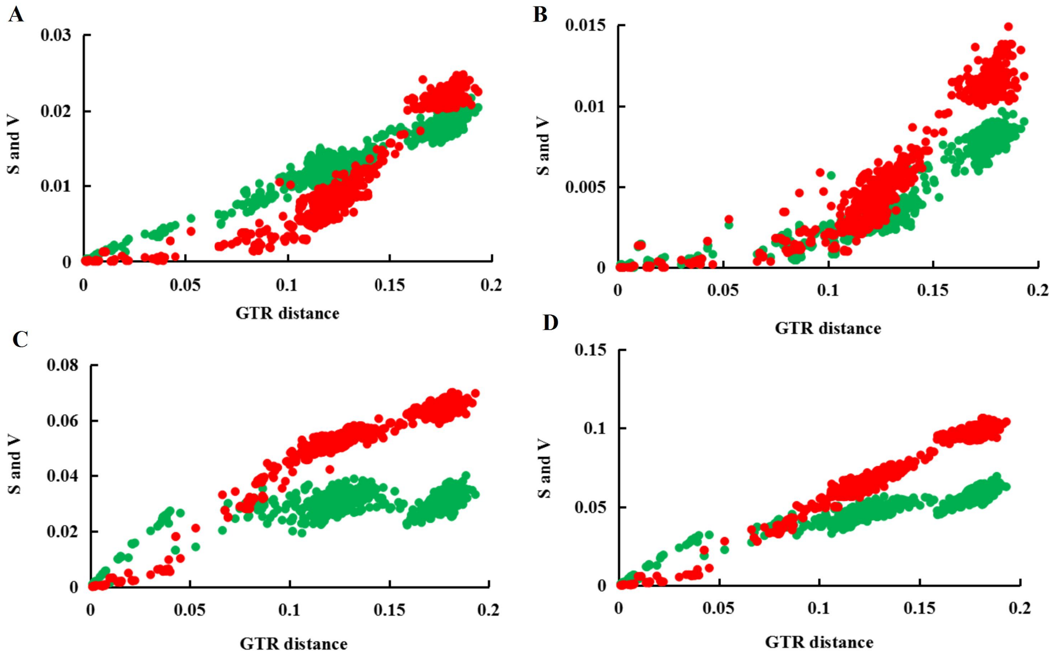

Supplementary Figure 4. Substitution saturation of 13 concatenated protein-coding genes (PCGs). Transitions (red) and transversions (green) were plotted against the GTR distance. A) First codon position of 13 PCGs; B) Second codon position of 13 PCGs; C) Third codon position of 13 PCGs; D) All sites of 13 PCGs.

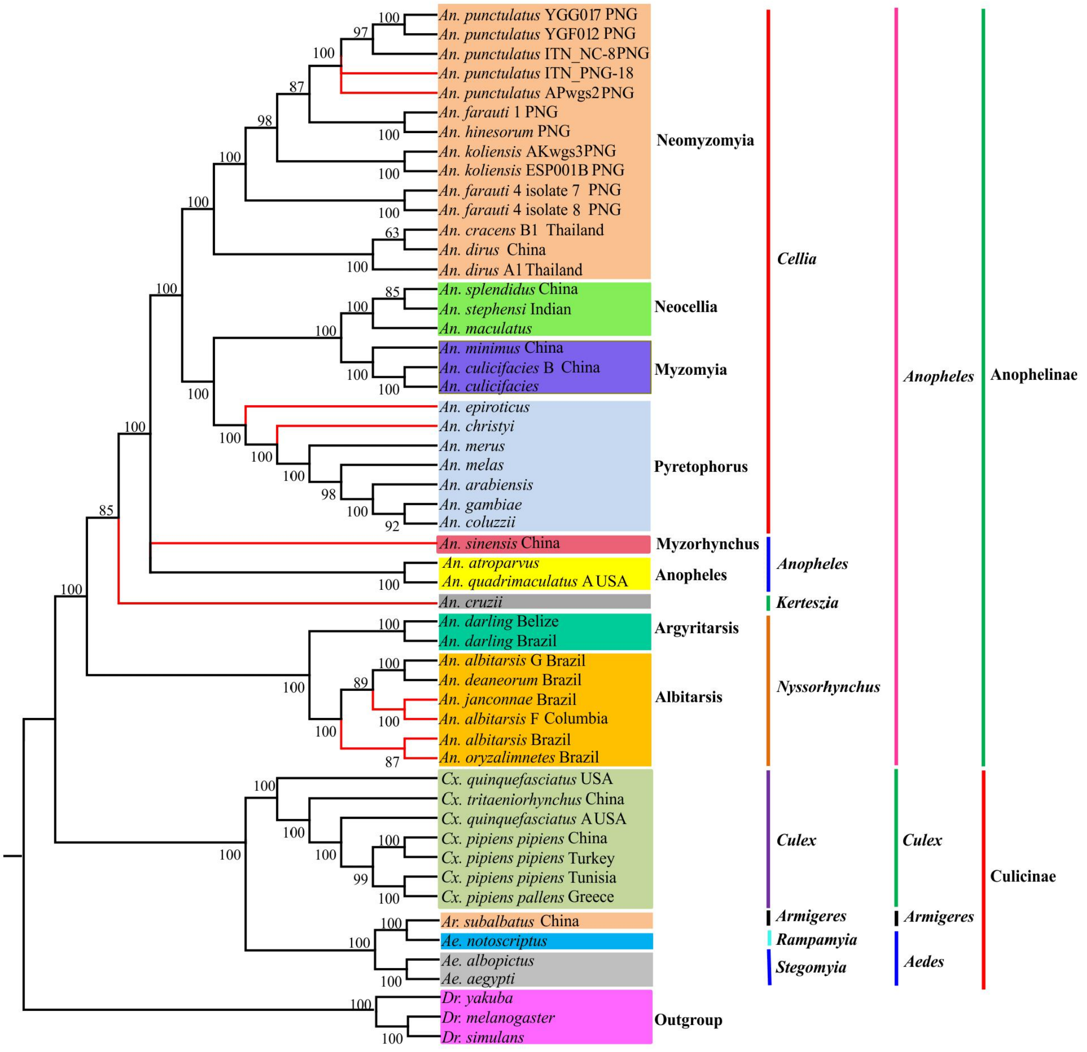

Supplementary Figure 5. Phylogenetic tree deduced by Bayesian-inference method based on the 1st and 2nd nucleotide of codons of the concatenated 13 PCGs of 50 mosquito mitogenomes. Bayesian posterior probabilities are shown for the corresponding branches. Branches in red indicate the different placing from the phylogenetic relationship inferred from PCG123.

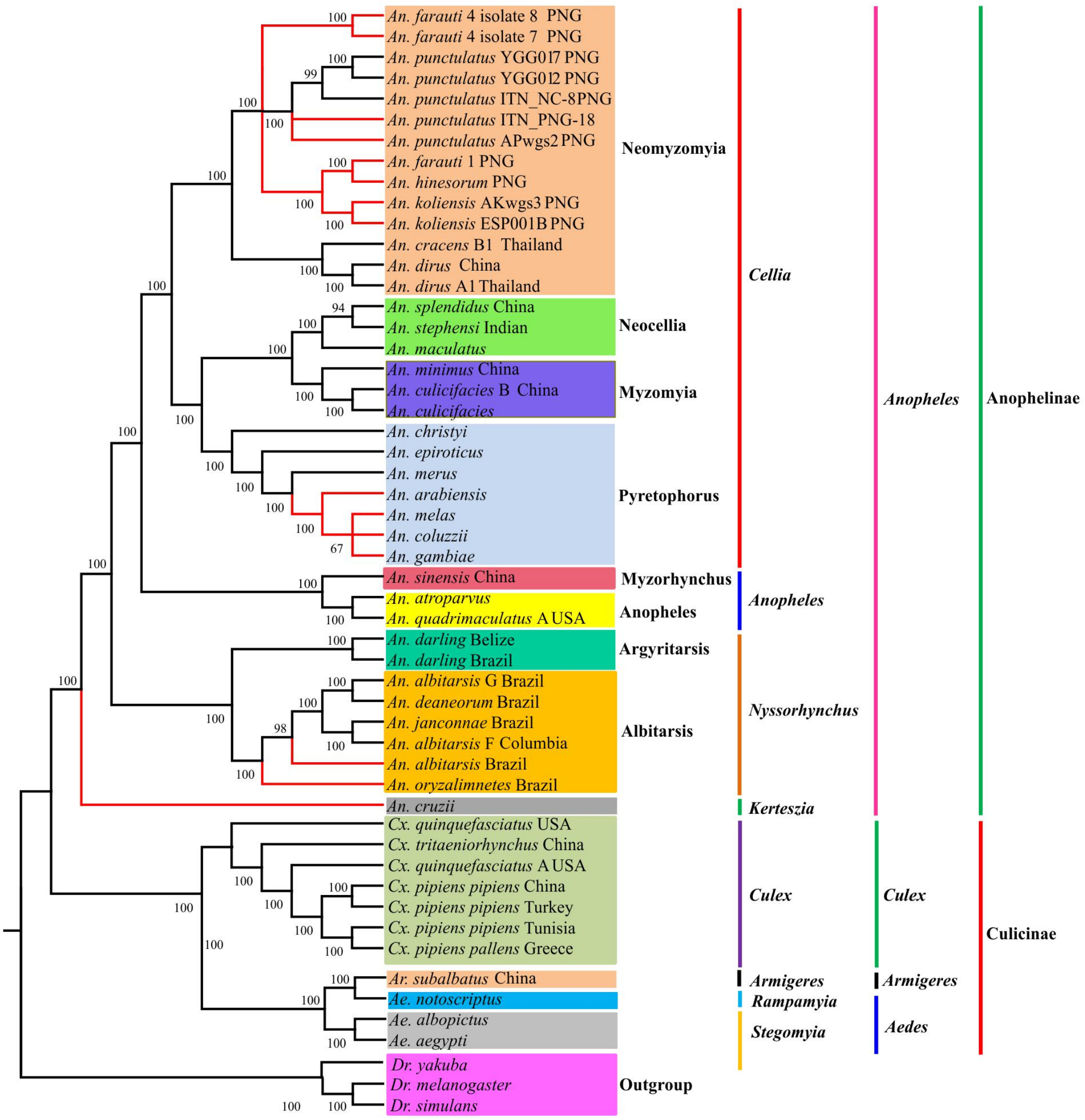

Supplementary Figure 6. Reconstruction of phylogenetic tree inferred by Bayesian inference method based on the concatenated 13 proteins of 50 mosquito mitogenomes. Bayesian posterior probabilities (BPP) are shown at relevant branches of the BI tree.

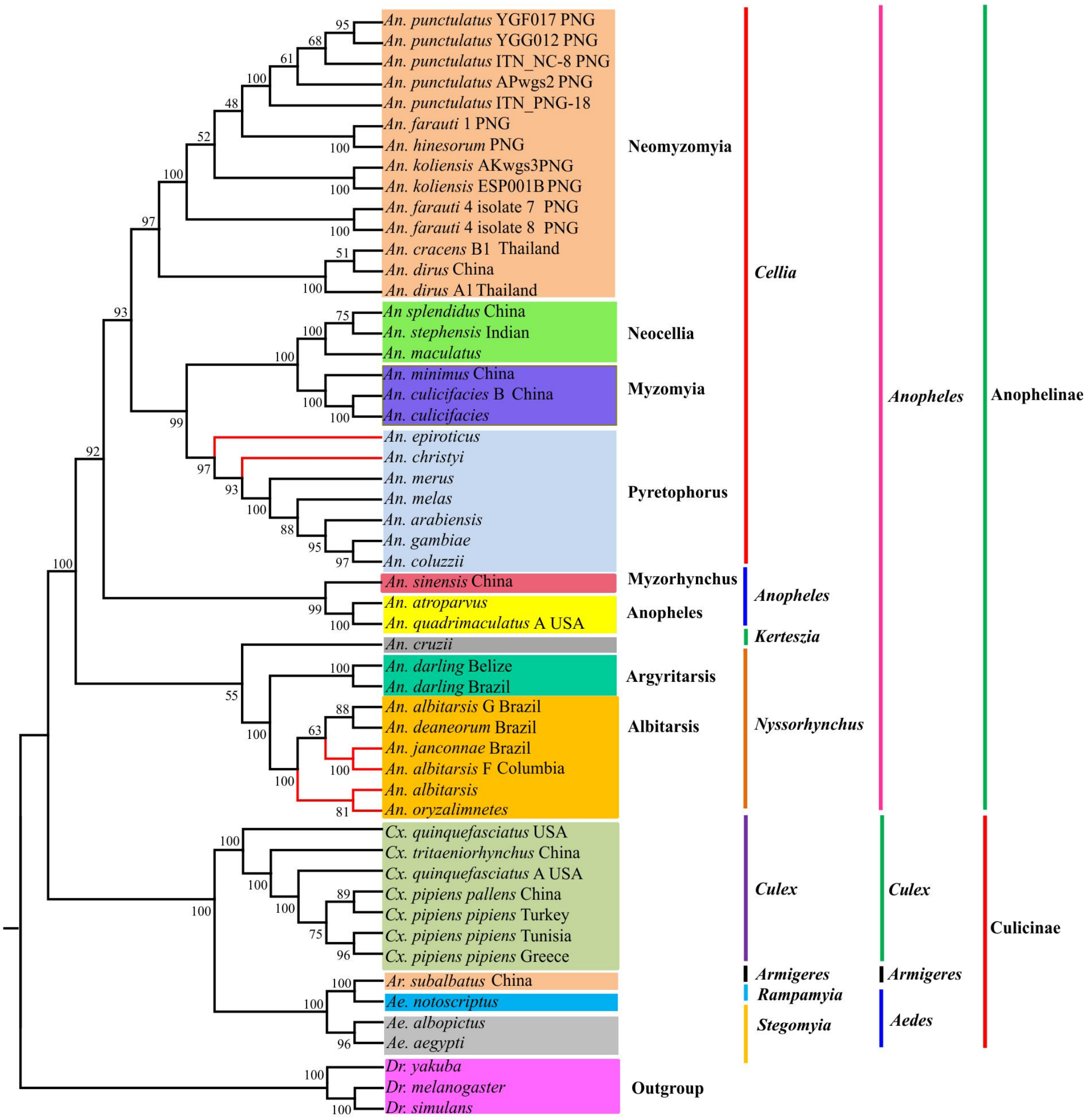

Supplementary Figure 7. Maximum-likelihood phylogenetic tree inferred by RAxML based on the 1st and 2nd nucleotide of codons (PCG12) of the concatenated 13 PCGs of 50 mosquito mitogenomes. Larger than 50% bootstrap support values from 1000 replications are shown on the corresponding branches. Branches in red indicate the different placing in the phylogenetic trees deduced from PCG12 and PCG123.

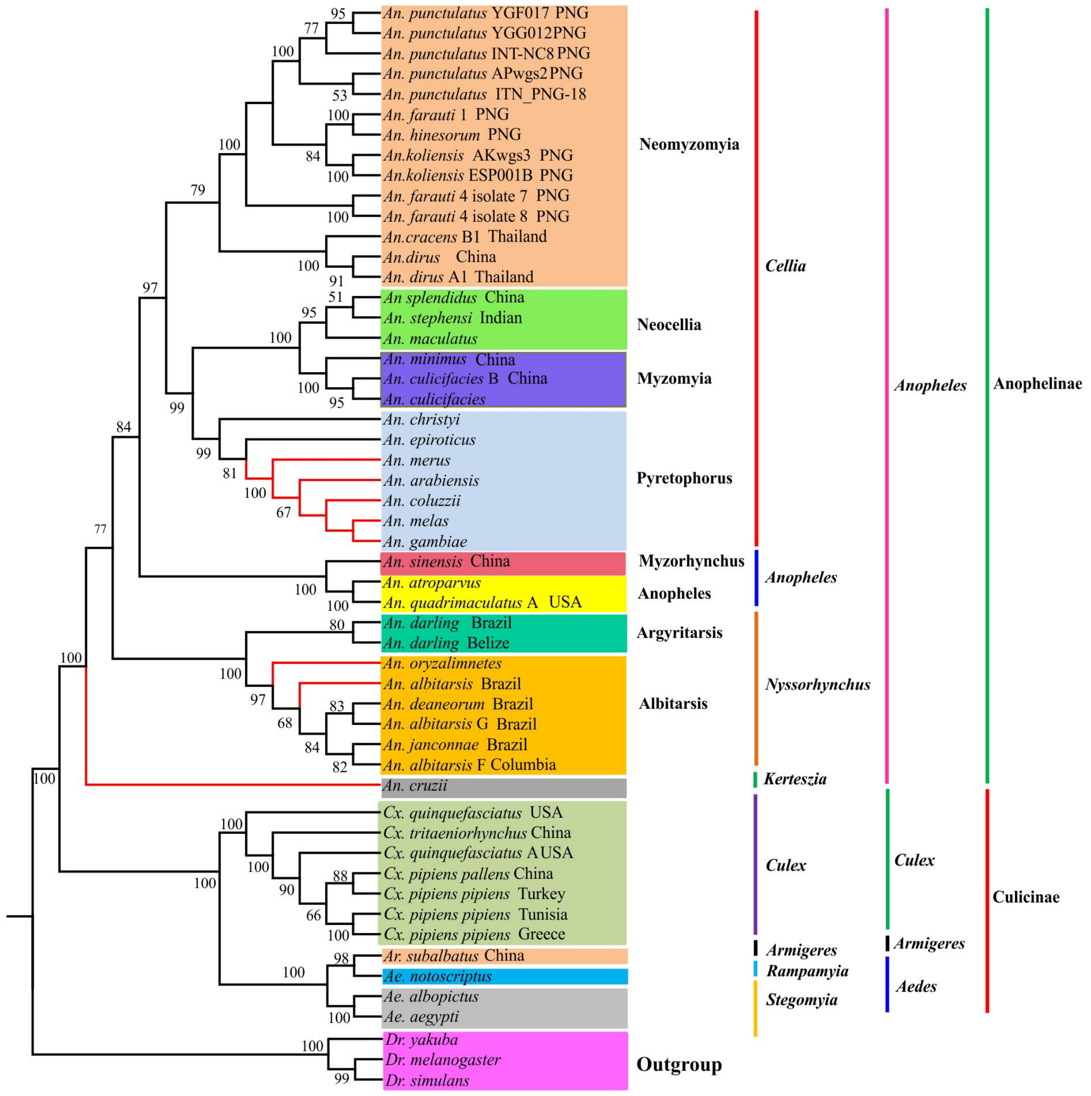

Supplementary Figure 8. Maximum-likelihood phylogenetic tree inferred by RAxML method based on of the concatenated 13 proteins of 50 mosquito mitogenomes. Bootstrap support values (BP) over 50% are shown on the corresponding branches. Branches in red indicate the different placing from phylogenetic relationship inferred from PCG123.
